# Development of Selection Indices for Improvement of Seed Yield and Lipid Composition in Bambara Groundnut (*Vigna subterranea* (L.) *Verdc*.)

**DOI:** 10.3390/foods11010086

**Published:** 2021-12-29

**Authors:** Razlin Azman Halimi, Carolyn A. Raymond, Bronwyn J. Barkla, Sean Mayes, Graham J. King

**Affiliations:** 1 Southern Cross Plant Science, Southern Cross University, Lismore, NSW 2480, Australia; razlin.mohd.azman.halimi@scu.edu.au (R.A.H.); carolyn.raymond@bigpond.com (C.A.R.); bronwyn.barkla@scu.edu.au (B.J.B.); 2 School of Bioscience, University of Nottingham, Loughborough LE12 5RD, UK; sean.mayes@nottingham.ac.uk; 3 Crops for the Future, NIAB-EMR, Cambridge CB3 0LG, UK

**Keywords:** underutilised crop, grain legume, selection indices, crop improvement, nutritional composition, seed lipid, plant protein

## Abstract

The underutilised grain legume bambara groundnut (*Vigna subterranea*) has the potential to contribute significantly to nutritional security. However, the lack of commercial cultivars has hindered its wider adoption and utilisation as a food source. The development of competitive cultivars is impeded by (1) lack of systematic data describing variation in nutritional composition within the gene pool, and (2) a poor understanding of how concentrations of different nutritional components interact. In this study, we analysed seed lipid and protein concentration and lipid composition within a collection of 100 lines representing the global gene pool. Seed protein and lipid varied over twofold with a normal distribution, but no significant statistical correlation was detected between the two components. Seed lipid concentration (4.2–8.8 g/100 g) is primarily determined by the proportion of oleic acid (r^2^ = 0.45). Yield and composition data for a subset of 40 lines were then used to test selection parameters for high yielding, high lipid breeding lines. From five selection indices tested using 15 scenarios, an index based on the seed number, seed weight, and oleic acid yielded a >50% expected increase in each of the mean values of seed number, pod dry weight, seed dry weight, and seed size, as well as an expected 7% increase in seed lipid concentration.

## 1. Introduction

Plant breeding programs aim to maximise the rate of increase in traits that are expected to have a genetic basis, where these traits can be inferred from data on the candidate lines under selection [1]. In private and public sector-breeding programs, prioritised traits often include high yield, together with contributing agronomic traits such as drought or heat tolerance, pest and disease resistance, and shorter maturation time [2,3,4]. The choice of traits under selection within breeding programs can also be determined by post-harvest market economics, such as the cultivation of specific wheat cultivars in Australia to meet the export market for Udon noodles [5]. In general, dietary nutritional traits of food crops have often had reduced priority compared with those affecting yield, appearance, and biotic resistance [6,7]. 

Underutilised crops face a set of interconnected challenges that hinder their wider utilisation as food sources. These challenges include poorly developed markets, neglect by research systems, together with fragmented and limited nutritional data [8]. Underutilised crops are increasingly being promoted as a means to counter reduced agrobiodiversity and nutritional security [9,10,11]. However, crop improvement that would expedite the transition from underutilised to increasing utility within food and farming systems is constrained by a lack of investment and coordinated research efforts [12,13,14]. 

The absence of high-yielding cultivars developed to suit different growing environments has been a major constraint for the adoption of the underutilised grain legume bambara groundnut (*Vigna subterranea* (L.) *Verdc*.) for food [15]. Bambara groundnut is a cleistogamous, self-pollinating, autogamous species. Although it displays high levels of inbreeding [16,17], few uniform cultivars have yet been developed [18,19]. The crop is closely related to cowpea (black eye pea; *Vigna unguiculata)* [20] and occupies the same agro-ecological niche as groundnut (peanut, *Arachis hypogaea* L.) in sub-Saharan Africa [21] and Southeast Asia [20,22]. However, it remains cultivated at the subsistence level [23], predominantly from farmers’ landraces [24,25] which are often a heterogeneous mix of several homozygous genotypes [26]. 

Breeding and line selection in bambara groundnut are conducted by research groups in Africa and Southeast Asia, with the local release of a limited number of varieties such as Mana and Kazuma in Zimbabwe (2004), Songkhla in Thailand (2010) and Nalbam 3, Nalbam 4, Nalbam 6 and Myao in Tanzania (2014) [18,19]. However, significant heterogeneity within the seed bulks of these ‘released varieties’ has been reported [27]. In addition, the history of modern selection is poorly documented, with most selective breeding efforts focusing on improving yield and drought tolerance within landraces without the benefit of genomic or marker-assisted selection [28,29,30,31]. Breeding is often limited by the availability of systematic multi-location, multi-year trialling data that may resolve issues such as yield instability [32] and photoperiod sensitivity [33]. To date, little emphasis has been placed on identifying traits that may predict yield stability [32], or on resolving the relative contribution of genotype (G), environment (E), and interactions (GxE) on yield traits [34]. Biomass growth rate, pod fill period, 100-seed weight, number of pods per plant, and time to flowering have been identified as traits that may play important roles in the improvement of grain yield and yield stability [32]. A critical review of the available nutritional data suggested that there is potential to increase seed protein and/or seed lipid concentration in bambara groundnut [35]. To date, studies quantifying nutritional components used materials sourced either directly from farmers or characterised traditional landraces obtained from markets [35]. However, little is understood about the relationship between yield and nutritional traits, including heritability estimates [36]. 

Selection indices (SI) are linear combinations of trait weightings and observed trait values that allow the simultaneous selection of multiple traits that may be otherwise correlated due to genetic interactions within breeding programs [37,38]. There are three types of linear selection indices (LSI) commonly used in plant breeding: phenotypic, marker, and genomic. Based on the first LSI proposed for plant breeding [39], up to 25 different variations of the three LSI types have been developed for use in plant breeding [1]. Selection indices have already been implemented into breeding efforts to improve yield in the minor legume pigeon pea (*Cajanus cajan* (L.) *Millsp*) [40,41]. However, to date, this useful pre-breeding tool has yet to be used for targeted improvement of yield and nutritional composition of bambara groundnut. 

The importance of plant seed-derived protein and oil in meeting the requirements of human and animal diets has driven the modern ‘re-domestication’ of crops such as canola rapeseed and soybean. These two components, along with starch, contribute to the available metabolizable energy derived from seed [42,43]. Lipid provides the highest per mass energy of 35−37 kJ/g, compared with protein (14.5−18.2 kJ/g) and total carbohydrate (10.4−17 kJ/g) [44]. The composition and distribution of fatty acids within the oil fraction determine the nutritional value, processing, storage, and cooking quality, flavour, and oxidative stability of the lipid [45,46]. Soybean, canola, and sunflower oil are regarded as having relatively high nutritional value due to elevated concentrations of monounsaturated fatty acids such as oleic (18:1, n-9), and the polyunsaturated fatty acids (PUFAs) linoleic (18:2, n-3) and linolenic (18:3, n-6) acid [46]. The existing literature indicates that in bambara groundnut seed, oleic acid (20–40%) is present in similar concentrations to that reported for soybean lines (20–35%) prior to improvement of oleic acid [35]. Previous studies based on analytical screening of germplasm have suggested there is a tenfold variation in seed lipid concentration (1–10 g/100 g seed) within the bambara global gene pool, compared with a fivefold for cowpea (1–5% seed) and sixfold for mung bean (1–6% seed) [35]. 

In this study, we established a global diversity set of 100 bambara groundnut lines (Table 1). In order to evaluate the potential of bambara groundnut as a good source of dietary lipid and unsaturated fatty acids, we performed de novo seed proximate and fatty acid analysis on the global diversity set. We then used a subset of 40 lines to evaluate how to yield components (seed weight, seed number, pod weight, pod number, shelling %), seed lipid, and unsaturated fatty acids (oleic acid, linoleic acid, linolenic acid) contributed as selection parameters for the development of pre-breeding SI. 

## 2. Materials and Methods

### 2.1. Seed Composition Dataset

#### 2.1.1. Sampling of Lines 

A global diversity set of 100 bambara groundnut lines from diverse geographical origins was established (Table 1). Seeds were sourced from Crops for the Future (CFF), Malaysia, the University of Nottingham (UoN), Sutton Bonington campus, UK, and the Australian Grains GeneBank (AGG). In total, 10 lines of soybean (*Glycine. max*), and cowpea (*V. unguiculata*) were sourced from AGG and provided by Prof. Terry Rose (Southern Cross University) and used as comparator species (Table 2). All seeds were ground to a fine powder using a Retsch ball mill with a 4 cm diameter stainless steel ball for two minutes at 30 Hz. Powdered samples were passed through a 450 µm sieve followed by a 300 µm sieve, and then re-ground in the ball mill for a further two minutes at 30 Hz. Samples were placed in Ziploc plastic bags and stored at 15 °C, 15% RH until use. 

#### 2.1.2. Compositional Analysis Method

Seed composition was analysed using the *Official Methods of Analysis* of the Association of Official Analytical Chemists (AOAC) International, 19th Edition, 2012 (Table 3). Moisture was quantified using gravimetric loss on the drying method (AOAC 925.09). Ash was quantified using the gravimetric loss on the ashing method (AOAC 942.05). Lipid was quantified using the gravimetric–Soxhlet method (AOAC 948.22) in an automated Gerhardt SOXTHERM^®^ (Germany) rapid extraction system for 90 min with n-hexane as the extraction solvent. Crude protein concentration was determined using the Dumas (horizontal combustion) method (AOAC 992.23) in a LECO TruMac Series Determinator (St. Joseph, MI, USA). A nitrogen-to-protein conversion factor of 6.25 was used for the calculation of crude protein. Total carbohydrate was calculated ‘by difference’—protein, lipid, ash, and moisture contents were determined and subtracted from the total weight of the sample [47,48]. Fatty acid composition of seed lipid was determined using the hydrolytic extraction gas chromatographic analysis of fatty acid methyl esters (FAMEs) method (AOAC996.06) in a Agilent 6890 Series Gas Chromatogram (Santa Clara, CA, USA) equipped with a Sephadex BPX70 capillary column (SGE054603; 50 mm × 0.22 mm ×1 µm film thickness) (Victoria, Australia) and Flame Ionised Detector (FID) (Santa Clara, CA, USA). The oven was set to 260 °C, EPC-Split Inlet set to 220 °C and 35.61 psi, helium flow at 258 mL/min with 200:1 split ratio and 255 mL/min split flow. Column pressure was set to 35.59 psi, helium flow at 1.3 mL/min, and velocity 29 cm/s. The FID heater was set at 300 °C, the H_2_ flow was set at 30 mL/min, the airflow was set at 350 mL/min, and the make-up flow (N_2_) was set at 25 mL/min. All analyses were performed in technical triplicate unless stated otherwise. Experimental data values (concentrations) obtained in g/100 g seed fresh weight were converted then to g/100 g of seed dry weight [49]. 

### 2.2. Selection Indices Dataset 

From the global dataset, a subset of 40 bambara groundnut lines, where agronomic data (yield) had been recorded in field trials over three years at one location, was selected for the development of selection indices (Table 1). The lines were planted at CFF’s Field Research Centre, in Semenyih, Malaysia (Latitude 2.931083, Longitude 101.878323 at 42 m above sea level), during dry planting seasons (December–April) in 2015, 2016, and 2017. The soil at the site is Ultisols (Rengam soil series, a clayey, kaolinitic, isohyperthermic Typic Paleudult) (Musa et al., 2016). Lines were planted in a randomised complete block design with 4 replicated blocks with 10 seeds planted per block for each line. Seeds were soaked overnight and treated with a fungicide prior to sowing. The experimental growing area was levelled and ploughed before each growing season, then ridges and furrows were constructed. One-metre-wide ridges were constructed, and the seeds were planted in two rows per ridge. For all lines, between-plant spacing of 40 cm and between-row spacing of 40 cm were maintained. Weed management was performed manually using hoe and hand pulling. Prior to planting, 60 kg/ha of phosphate (P_2_O_5_) and 60 kg/ha of potassium (K_2_O) using urea (46% N) and muriate of potash (60% K_2_O) were mixed thoroughly with the soil. Nitrogen fertiliser was applied at sowing at 20 kg/ha. Watering was maintained at 50–70% field capacity until flowering. Earthing up was performed twice—at flowering and at the onset of pod formation. Plants were harvested at maturity using a hand hoe, followed by threshing, shelling, and oven drying the pods at 35 °C for 7 days. Pods and seeds were weighed, sealed in paper bags, and stored in DryStore^®^ system low humidity storage barrels set at 10% RH until use.

The following traits were recorded over three successive years of field trials: days to emergence, days to flowering, days to podding, seed number, seed dry weight, and pod dry weight. Five agronomic traits were selected for use in the selection indices: seed number, pod dry weight, seed dry weight, single seed size, and shelling percentage. All in-field traits are standard measurements according to the International Plant Genetic Resources Institute (IPGRI) descriptors for bambara groundnut [50]. Single seed weight and shelling percentage were calculated using the following formula:

$$\mathrm{Sin}\mathrm{gle}\;\mathrm{seed}\;\mathrm{size}=\frac{\mathrm{Weight}\;\mathrm{of}\;\mathrm{all}\;\mathrm{seeds}\;\times 100\%}{\mathrm{Total}\;\mathrm{number}\;\mathrm{of}\;\mathrm{seeds}}$$

$$\mathrm{Shelling}\;\mathrm{percentage}=\frac{\mathrm{Total}\;\mathrm{weight}\;\mathrm{of}\;\mathrm{seeds}\;\times 100\%}{\mathrm{Total}\;\mathrm{weight}\;\mathrm{of}\;\mathrm{pods}}$$

where possible, we included controlled vocabularies from the Crop Dietary Nutritional Ontology (CDNO) [51] and bambara groundnut Crop Ontology (CO_366) [52] for the nutritional (seed composition) and agronomic traits used in this study (Table 3).

### 2.3. Statistical Analysis 

All analyses were performed using GenStat 64-bit version 19.1 (VSN International Ltd., Hertfordshire, UK) software. For seed composition data, analysis of variance (ANOVA), and least significant difference (LSD at 5% level of probability) for comparisons were determined. Principal component analysis (PCA) on correlation matrix was performed on lipid and fatty acid data. All subset regression analysis and multiple linear regression models were used to explore important inter-relationships within the seed composition data and within the lipid and fatty acid data. Regression analyses were plotted using the scatterplot function and fitted with linear trendlines in Excel^TM^. A between-trait correlation matrix was performed on the selection index dataset (*n* = 40) and then used as input for a PCA. Correlations were considered statistically significant at *p* < 0.05 if r > 0.312 (*n* = 40, degrees of freedom (d.f) = 38) [53].

### 2.4. Heritability Analysis 

Two methods were used to estimate the broad-sense heritability (genetic control) of each trait used for the selection indices. The first method used parent–offspring regression analysis on six lines (Ex-Sokoto-26, Kaaro 4, Kaaro-74, Songkhla-1, IITA686_CFF, and Burkina) grown for three successive years in the same plot at CFF (indicated with an asterisk (*) in Table 1). Linear regression lines for parent and offspring were plotted using the scatterplot function in Excel^TM^. The R-square value for each regression line was used as the heritability estimate. The second method involved a small GxE study on twelve lines, (DodR_CFF, DodR_UoN, IITA686_CFF, IITA686_UON, S19-3_CFF, S19-3_UoN, Uniswa Red_AB_CFF, Uniswa Red-Red_UoN, DipC_CFF, DipC_UoN, Gresik_CFF, and Gresik_UoN) representing six lines grown in two environments (UoN, UK and CFF, Malaysia) (indicated with double asterisks (**) in Table 1). The variance components (VC) for genotype (G), environment (E), their interaction (GxE) plus residual (R) were estimated using restricted maximum likelihood (REML) analysis. Heritability was estimated using the following formula:

$$\mathrm{Heritability}=\frac{\mathrm{Genotype}\;\mathrm{VC}}{\left(\mathrm{Genotype}\;\mathrm{VC}+\mathrm{GxE}\;\mathrm{VC}+\mathrm{Residual}\;\mathrm{VC}\right)}$$

Trait heritability was estimated as an average of heritability using the two methods.

### 2.5. Selection Index and Scenario Testing

A total of 5 selection indices were developed, and 15 scenarios were tested within the indices (Table 4). The following traits were selected for the indices: seed lipid, seed protein, seed carbohydrate, oleic acid, linoleic acid, linolenic acid, seed number, pod dry weight, seed dry weight, seed size, and shelling percentage. To standardise the trait data, all values were converted to a common scale of standard deviation (standard normal deviate, SND) units using the following formula:

$$\mathrm{Standard}\;\mathrm{normal}\;\mathrm{deviate}=\frac{\left(\mathrm{Mean}\;\mathrm{of}\;\mathrm{line}-\mathrm{mean}\;\mathrm{of}\;\mathrm{database}\right)}{\mathrm{Standar}\;\mathrm{deviation}}$$

Scenario scores were calculated as a sum of the trait weighting multiplied by the SND value for all traits selected. The indices differed in the relative importance ascribed to each trait. In each scenario, selected traits were given a weighting of two or three, while traits not selected were given a weighting of one. A weighting of three was assigned to the highest value traits of seed lipid, oleic acid, linoleic acid, and linolenic acid, and a weighting of two was given to seed number, seed dry weight, and seed size (Table 4). Independent culling levels were applied to the output to prevent traits from falling below acceptable limits. Bambara groundnut lines falling below the following thresholds were eliminated as potential candidates for further breeding: (1) seed protein below 15 g/100 g seed dry weight and (2) shelling percentage below 50%. Lines with positive scores for each scenario in each index were compiled. The predicted % of change, in original measurement units, for all traits (seed number, pod dry weight, seed dry weight, seed size, shelling %, seed lipid, protein, carbohydrate, linoleic acid, oleic acid, and linolenic acid) from the mean value for each index was then calculated using the following formula:

$$\%\;\mathrm{change}=\frac{\left(\mathrm{Mean}\;\mathrm{of}\;\mathrm{top}\;15\;\mathrm{positive}\;\mathrm{scores}-\mathrm{mean}\;\mathrm{of}\;40\;\mathrm{lines}\right)\times 100\%}{\mathrm{Mean}\;\mathrm{of}\;40\;\mathrm{lines}}$$

## 3. Results

**Figure 1:**
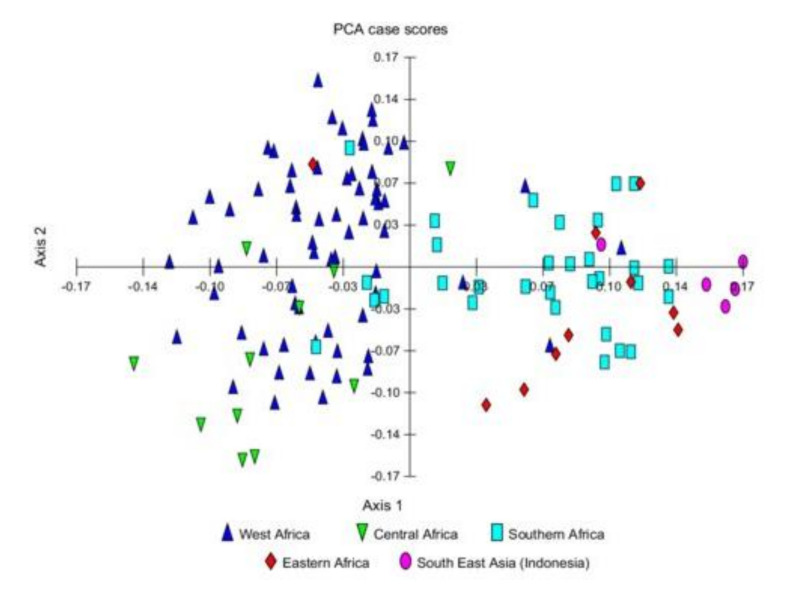
Principal coordinate analysis (PCA) scatter plot for 123 bambara groundnut landraces from Africa and Southeast Asia (Indonesia) based on 12 SSR markers used to determine population structure. Used with permission from [56].

We established a set of 100 lines (Table 1) representing the global bambara groundnut gene pool from diverse geographical origins of sub-Saharan Africa (south, east, west) and Southeast Asia (Indonesia and Thailand). The dataset represents an estimated 2% of the accessions conserved in ex situ collections globally (4500 accessions) [20]. These lines have had no known quantified or documented intentional selection pressure for nutritional improvement. Previous studies have established that in populations with allelic frequencies > 2%, a global gene pool collected from natural populations should contain 99% of the allelic polymorphism [54,55]. Analysis of bambara population structure [56] indicated two main grouping of lines based on geographical origins with two main sub-groupings: west African and central African accessions, denoted as population sub-group one, and southern African, eastern African, and the Southeast Asian accessions, clustered together as the second group (Figure 1). 

### 3.1. Compositional Analysis

**Figure 2:**
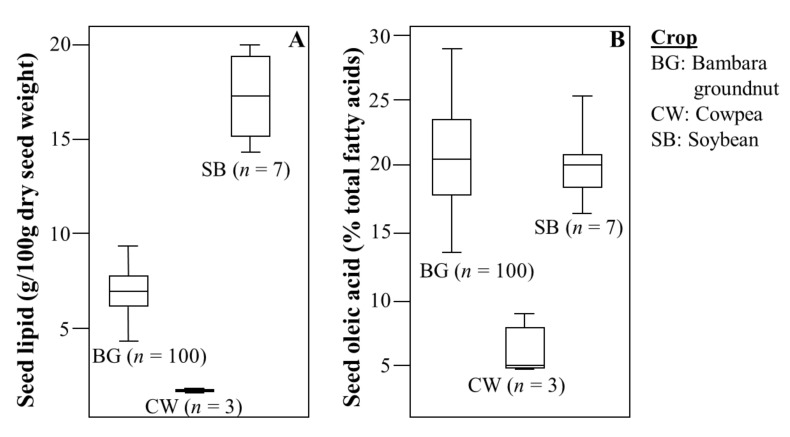
Box-whisker plots indicating (**A**) range of variation for seed lipid concentration and (**B**) seed oleic acid concentration (18:0, n-9) in the global diversity set of 100 bambara groundnut (BG) lines and in representative lines of two other grain legumes: cowpea (CW; *n* = 3), and soybean (SB; *n* = 7). For each crop, number of lines (*n*) analysed is indicated below each corresponding box whisker. Y axes: (**A**) seed lipid expressed as g/100 g seed dry weight and (**B**) seed oleic acid expressed as % of total fatty acid.

The distributions of concentration for seed macronutrients (carbohydrate, protein, lipid, and dietary fibre) determined for the global diversity set of 100 bambara groundnut lines (Supplementary Table S1) indicated significant variation between lines (*p* < 0.01), with minor variation (standard deviation, SD = 0.00 to 0.92, ANOVA) between technical triplicates, and a normal distribution of values across the gene pool. Seed carbohydrate for bambara groundnut varied between 58.7 and 70.0 g/100 g dry weight. (Supplementary Table S1). Seed lipid ranged from 4.2 to 8.8 g/100 g dry weight, with a maximum value six times that observed in cowpea (1.3−1.7 g/100 g) (Figure 2A). Seed protein ranged between 14.6 and 28.9 g/100 g dry weight, a much wider range than that obtained for cowpea (24.0−28.5 g/100 g), although the highest values were similar. In comparison, values for soybean seed lipid (15.3−21.5 g/100g dry weight; Figure 2A) and protein (40.7−50.3 g/100 g dry weight) were twice those observed for bambara groundnut (Supplementary Table S1). No significant relationship was detected between seed protein and lipid concentrations (r^2^ = 0.004) or between seed lipid and carbohydrate concentrations (r^2^ = 0.08). However, a weak negative relationship was observed between seed protein and carbohydrate concentration (r^2^ = −0.40, *p* < 0.05). Within the bambara lipid fraction, linoleic acid (18:2 n-6) accounted for 33−45% of seed oil, oleic acid (18:1, n-9) 15−29%, and palmitic acid (16:0) 16−23% (Supplementary Table S2). The distribution of oleic acid concentration within the 100 bambara groundnut lines was similar to that observed for seven soybean lines (18−26%) (Figure 2B). A PCA of the correlation matrix for seed lipid and eight fatty acids (oleic, linoleic, palmitic, stearic, arachidic, behenic, lignoceric, and α-linolenic acids; Supplementary Figure S1) indicated a possible positive relationship between seed lipid and three fatty acids: oleic, lignoceric, and behenic acids. Regression analysis showed that oleic acid had the strongest positive correlation to seed lipid (r^2^ = 0.45, *p* < 0.01). A significant negative relationship between oleic acid and linoleic acid (r^2^ = 0.58, *p* < 0.01) was also observed. Regression analyses also showed a significant negative correlation between α-linolenic acid and oleic acid (r^2^ = 0.55, *p* < 0.01), and a positive but weaker association between α-linolenic acid and linoleic acid (r^2^ = 0.44, *p* < 0.01). 

### 3.2. Development of Selection Indices

A base linear phenotypic selection index (BLPSI) was applied for this study due to a lack of available estimates for the genetic parameters and economic weights relating to bambara groundnut traits. A total of 5 phenotypic SI with 15 scenarios were tested. Within the scope of developing the SI, we also performed a pilot analysis of broad-sense heritability using two small subsets of lines. The results allowed us to identify specific bambara groundnut lines that could contribute as pre-breeding material to increase yield, lipid, and unsaturated fatty acid concentration.

**Figure 3:**
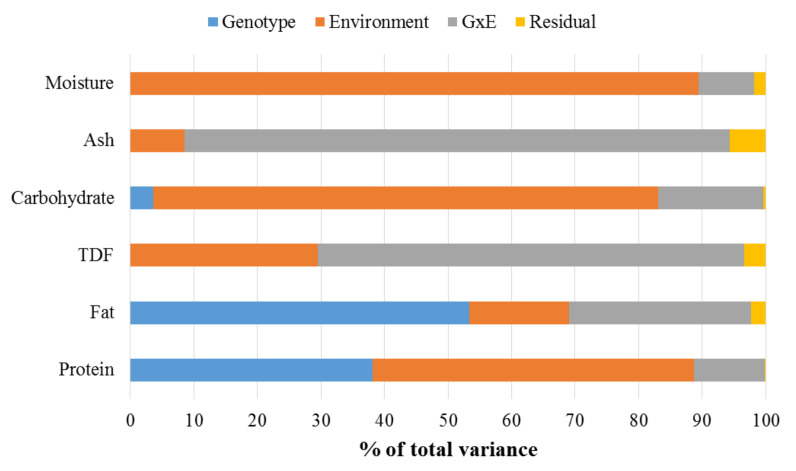
Proportions of total variation and the relative contribution of genotype (G-blue), growing environment (E-orange), and their interactions (GxE-grey) to proximate nutritional components (moisture, ash, carbohydrate, total dietary fibre (TDF), lipid, and protein) using six bambara groundnut lines grown in Malaysia and United Kingdom. Variance components for genotype, environment, GxE, and residual were estimated using restricted maximum likelihood (REML) analysis.

Within the dataset used for the development of SI, PCA of the correlation matrix for the five agronomic traits (seed number, pod dry weight, seed dry weight, single seed size, shelling %) and the seven nutritional traits (lipid, protein, carbohydrate, oleic, lignoceric, linoleic, and linolenic acids) (Supplementary Figure S2) indicated that PC1 accounted for 46.3% and PC2 18.3% of the variation. Within PC1, seed number, seed dry weight, pod dry weight, single seed size, protein, lipid, oleic acid, and lignoceric acid were positively loaded, while carbohydrate, linoleic acid, linolenic acid, and shelling % were negatively loaded (Supplementary Figure S2). Correlation analysis (Table 5) showed significant positive relationships between the four yield traits of seed number, pod dry weight, seed dry weight, and single seed size at *p* < 0.05. The shelling percentage was negatively correlated with the other four yield traits. Oleic and lignoceric acids had significant positive relationships with seed lipid and the four yield traits, while linoleic and linolenic acids were negatively correlated. Heritability estimates (Table 6) indicated that nutritional traits had higher heritability in comparison with agronomic traits. Variation in seed lipid (>50%) is mainly attributed to genotype, while seed protein variation (>50%) is mainly attributed to the environment rather than genotype (Figure 3).

A set of 11 lines had positive scores for all scenarios tested in the following indices SI-1(oleic acid), SI-3 (oleic acid and yield), and SI-5 (yield) (Table 7). The five lines (IPB-Bam2, IPB-Bam1, GHC36105, 99SB42-NAM-C, and BD) with the highest scores in each scenario tested in the three SIs shared common agronomic and nutritional characteristics. The five lines had larger seed size (0.7−0.9 g per seed), dark colour seed coat (black, brown, dark red), >7% seed oil with high oleic acid concentration (20−25%), but low linoleic acid concentration (33−36%). 

To evaluate the functionality of the indices as a pre-breeding tool to identify potential breeding lines to improve the yield and nutritional composition, the predicted % change over the mean values for each trait (seed number, pod dry weight, seed dry weight, seed size, shelling %, lipid, protein, carbohydrate, linoleic acid, oleic acid, and linolenic acid) was calculated. Using either SI-1 or 3, there was a predicted increase of more than 50% in seed number, seed dry weight, and pod dry weight, a 7–8% increase in seed lipid, and ~9% increase in oleic acid (Table 8). If the bambara groundnut lines were selected using parameters set for SI-1 rather than SI-3, seed lipid concentration would be increased, but the yield components would be 10% lower. The selection of bambara groundnut lines using either SI-1 or SI-3 predicted an increase in seed protein of 2–4%, but a decrease in seed carbohydrate of 1–2% (Table 7). Selection of lines based either on omega 3 and 6 fatty acids (SI-2) or on omega 3 and 6 fatty acids and yield (SI-4) was predicted to decrease both seed lipid and yield (Table 7). SI-5 was found to contribute the highest increase in seed number, pod dry weight, and seed dry weight, and ~5% increase in seed lipid. 

## 4. Discussion

This study is the first significant effort to evaluate the nutritional composition of a set of 100 bambara groundnut lines representing the geographical distribution within the genetic centres of diversity in sub-Saharan Africa and Southeast Asia. There were some confounding effects of genotype × location × year on the evaluation of nutritional composition, due, in part, to constraints in terms of seed quantity and quality, and quarantine restrictions for seed imports. The dataset, therefore, includes seeds from lines grown in different locations, with the variance attributable to each environment unknown. For seed lipid, variance due to genotype exceeded variance due to environment, based on the pilot GxE analysis of a subset of six lines grown in the UK and Malaysia (Table 6). For seed protein, variance due to environment was more prominent (Figure 3), and for linoleic and oleic acid, variance due to environment was approximately three times greater than genotype. However, for oleic acid, the variance due to environment and genotype were similar (40% and 35%, respectively). Similar constraints have been reported for studies in soybean [57,58,59] and cowpea [60,61,62]. To date, there have been few, if any, detailed genetic studies to determine genetic, non-genetic, or GxE interactions for any trait in bambara groundnut. This includes a lack of accurate heritability estimates and expected gain from the selection. However, a GxE study based on 40 bambara groundnut lines [36] indicated that, as expected, nutritional traits such as seed protein concentration have stronger genetic variance than agronomic traits such as pod yield. More extensive and systematic multi-location, multi-year studies using starting materials regenerated from a single environment are required to increase the resolution of variance estimates. For the SI dataset used in this study, although there was no replication, we believe that there was no carry-over from the geographical origin of the lines since the seeds used were sourced from plants grown in three generations in the same location (CFF, Semenyih, Malaysia).

### 4.1. Compositional Analysis 

Previous studies based on a limited sampling of the gene pool suggested there may be valuable variation in nutritional composition within the bambara groundnut gene pool [35]. Our more extensive analysis of a global diversity set based on quantification in a single laboratory indicates that variation observed within the bambara groundnut gene pool is consistent with distributions described for other grain legume species, with high protein (20−25% of seed) and carbohydrate (50−65%), and low lipid (<10% of seed) [63,64,65,66]. Total seed lipid had a normal distribution, with significant variation between lines -(*p* < 0.01, ANOVA), ranging from 4.2 g/100 g dry weight for the line Mottle Black to 8.8 g/100 g dry weight for the line BD. Within the diversity set, 12 lines (12%) had seed lipid higher than 8.0 g/100 g dry weight. Seed lipid concentration in the BD line was almost six times the concentration for the cowpea lines used in this study, but only half of the value for the soybean lines [59,67] (Figure 2A). For the high lipid (>8 g/100 g seed) bambara groundnut lines, the lipid was calculated as contributing 16−18% of total metabolised energy, with the remainder accounted for by carbohydrate and protein. Due to the higher food energy conversion factor of lipid (35 kJ/g), compared with carbohydrate (17 kJ/g) and protein (12 kJ/g), increasing seed lipid would increase the total per mass metabolised energy, although there may be a corresponding yield penalty which has yet to be determined for bambara groundnut. In soybean breeding, several high lipid lines having high oleic acid (>60% of fatty acids) and stable seed yield have been developed [68,69]. Similar outcomes have been achieved in modern peanut cultivars [70], and in hemp, where modern oilseed cultivars have been developed with 70 molar percent oleic acid [71]. 

Based on our analysis and previous literature survey [35], we propose that increasing seed lipid concentration in bambara groundnut would be a viable strategy to enhance the value of this crop for human consumption. The value would be added both by increasing the overall energy intake and through the increased ingestion of monounsaturated fatty acids such as oleic acid. The genetic logistics of this strategy is supported by regression analysis, which showed that there are no statistically significant trade-off relationships between seed protein and lipid (r^2^ = 0.004), nor between seed lipid and carbohydrate (r^2^ = 0.08). However, there was a weak trade-off relationship between seed protein and carbohydrate (r^2^ = 0.40, *p* < 0.05). It also appeared that seed lipid and oleic acid were positively correlated with yield traits such as seed number, seed dry weight, pod dry weight, and single seed weight (Table 5). Similar positive relationships between seed yield components, seed lipid, and oleic acid have been observed in soybean [45,69,72,73] and peanut [70,74]. Although this correlation holds true for the subset of 40 bambara lines used in this study, there may be deviations for other subsets of lines grown under different environments. A more extensive multi-location study would be required to establish the consistency of trade-offs between the agronomic and nutritional traits. 

### 4.2. Development of Selection Indices

The main aim of this study was to develop selection indices as a pre-breeding tool to increase yield, seed lipid, and unsaturated fatty acid concentration in bambara groundnut. In major legumes such as chickpea (*Cicer aritinum*), SIs have been predominantly used to breed high-yielding and drought-resistant cultivars [75,76,77]. In common bean (*Phaseolus vulgaris*), SIs have been used to identify superior genotypes for yield, grain size, disease resistance, and cooking time [78,79] and to predict increases in genetic gain for selected traits in multigenerational families [80]. Within the *Vigna* genus, SIs have been applied to cowpea breeding in Brazil [62], to identify superior genotypes that fulfilled a set of agronomic (days to flowering, days to maturity, pod length, pod number, 100 seed weight, and grain yield), nutritional (protein, iron, and zinc), and culinary traits (cooking time). Five SIs were developed, and fifteen scenarios were tested with varied combinations of traits and trait weighting (Table 4). Independent culling levels set at 15% seed protein and 50% shelling were applied to the output to prevent traits falling below acceptable limits. The recommended daily intake (RDI) of protein for children is set at 14 g/day for ages 1−3 and 20 g/day for ages 4−8 [81]. In many subsistence farming communities where bambara groundnut is cultivated, a 100–200 g serving of bambara groundnut would provide sufficient protein to combat protein malnutrition in children. In legumes, shelling percentage is the second most important agronomic trait after pod yield, as it represents the ratio of seed to combined (pod + seed) weight [82]. A higher shelling percentage indicates a more edible portion (seed) and less waste (shell or pod). In peanut cultivars, this trait significantly affects the economic value of the cultivar and can vary from 45.3% to 81.0%, within a breeding population [83,84]. Within the selection index dataset used in this study (*n* = 40), shelling % varied from 29 to 81%. This variance is greater than reported for other smaller subsets of bambara groundnut. For example, 12 genotypes grown in Malaysia had a shelling percentage between 40 and 78% [85] and a subset of 22 landraces evaluated in Ghana had reported a shelling percentage of 46−74% [86]. A survey of farmers and small-scale processors in Zimbabwe indicated a preference for a minimum 60% shelling percentage in bambara groundnut [87]. 

Eleven of the 40 bambara groundnut lines had positive scores for all scenarios tested in the three selection indices, seed lipid (SI-1), seed lipid and yield (SI-3), and yield (SI-5) (Table 7). The set of 11 lines encompasses 2 groups based on geographical origin: sub-Saharan Africa and Southeast Asia. In general, the lines from sub-Saharan Africa had cream or light brown testa, while the lines from Southeast Asia had darker (black, dark purple) testa colour. These are in line with farmers’ seed preferences in sub-Saharan Africa [88,89] and Indonesia [90,91]. Light-coloured seeds fetch premium prices in markets in sub-Saharan Africa due to higher demand in comparison with red and black testa seeds [89]. In addition, sensory evaluation of bambara groundnut ‘milk’ (similar to aqueous extracts marketed as almond ‘milk’) indicated that milk made with lighter-coloured seeds was more acceptable to the taste panel [92]. Inclusions of end-user preferences for seed size, colour, or taste within SI are important considerations for bambara groundnut breeding programmes, as these traits play pivotal roles in acceptance of the lines by farmers [20,86,93]. In addition, ‘traits’ such as hard to cook and anti-nutritional factors could also be used selection parameters for future SIs, as they have been shown to affect digestibility, nutritional quality, and acceptance of bambara groundnut as food [28,93,94,95]. 

For SI-1 and SI-3, an increase of >50% to seed number, seed dry weight, and pod dry weight, 7−8% increase in seed lipid, and ~9% increase in oleic acid was expected for the lines selected (Table 8). If the bambara groundnut lines were selected using the parameters set for SI-1, a higher % increase in seed lipid would be obtained, but the expected % of change to yield components would be ~10% lower in comparison with using parameters set for SI-3. Selection of bambara groundnut lines using either SI- 1 or SI-3 is expected to increase seed protein by 2−3% but decrease seed carbohydrate by 1−2% (Table 8). This will not affect the metabolised energy content (kJ/100g) of the lines, as on a per gram basis lipid contributes more energy (35 KJ/g) in comparison with protein (14.5 KJ/g) and carbohydrate (17 KJ/g) [49]. Based on the analysis in this study, SI-3 was identified as the most suitable index for the selection of high-yielding, high-lipid bambara groundnut lines, originating from arid sub-Saharan Africa or humid Southeast Asia. Once the suitable lines are selected using the SIs, the oleic acid content of these lines could then be genetically enhanced using similar approaches to those used in soybean breeding. 

In soybean, cultivars with 60−80% oleic acid have been developed [45] from conventional soy germplasm that initially had 25% oleic acid [96,97], which is similar to the values for bambara groundnut and obtained in this study (15−29%; Figure 2B). Improvements in oilseed lipid concentration by manipulation of seed oleic acid are common in species such as soybean [45,98] and groundnut [70]. Increasing oleic acid also increases long-chain unsaturated fatty acids, perceived as beneficial to the human diet. Increasing seed oleic acid improves the oxidative stability of the seed lipid fraction [45] in storage and has been shown to lower low-density lipoprotein (LDL) cholesterol in humans [68]. Therefore, we propose that an approach similar to that used for soy could be used to increase the bambara groundnut seed oleic acid and lipid concentration. However, the significant negative correlations between seed oleic acid and linoleic acid (r^2^ = 0.58) and between seed oleic acid and linolenic acid (r^2^ = 0.55) would need to be considered. We were interested in exploring the scope for achieving similar outcomes in bambara groundnut. Increasing oleic acid content in bambara groundnut from 25 to 45% was calculated to increase seed lipid by 1.2%. For major crops such as soybean and groundnut, the increase was accomplished primarily by combining alleles [70,99] that encoded modifications to the FAD2 family of genes that encode the ω-6 desaturase enzyme, which is responsible for desaturation of linoleic to oleic acid [100]. Similarly, targeted mutations of genes encoding FAD2 desaturase enzymes (*csfad2a-1*) have resulted in oilseed hemp varieties with 70 molar percent higher oleic acid [71]. To the best of our knowledge, there has been no effort to identify FAD genes in *Vigna* crops such as cowpea or mung bean, as these typically have low seed lipid [101,102]. However, research effort has focused on improving seed protein [103] and mineral composition [104]. 

## 5. Conclusions

Understanding sources of variation in crop-derived dietary components is a key factor that can contribute to the development of competitive cultivars developed for different growing environments. We carried out the first systematic evaluation of variation and interactions in seed macronutrients and lipid fraction for bambara groundnut based on a representative coverage of the gene pool. This study is also the first to evaluate the feasibility of developing pre-breeding selection indices to optimise yield and nutrition. In total, 5 simple selection indices were tested in 15 scenarios. Weightings for each trait were assigned according to results from PCA), correlation analysis, and heritability analysis. Including additional lines in the future will provide better estimates of heritability for yield and nutritional traits. Further research is also needed to establish the genetic parameters within bambara groundnut for the traits so that more sophisticated selection index equations incorporating genomic selection can be utilised.

Seed lipid, omega 3 and 6 fatty acids, and yield parameters (seed weight, pod weight, seed number, shelling percentage) were the main parameters tested within the selection indices. SI-1 and SI-3 appeared to be the most favourable for selecting high-yielding, high-lipid Bambara groundnut lines (Table 8). Trade-offs were detected in different selection indices that may guide future breeding efforts in different regions.

## Figures and Tables

**Table 1:** List of bambara groundnut lines used to construct global diversity dataset for de novo analysis of seed proximate composition (indicated with SC), and subset of 40 lines used for development of selection indices (indicated with SI). Lines labelled with asterisk (*) were used for broad-sense heritability estimation using parent-offspring regression. Lines labelled with double asterisks (**) were used for broad-sense heritability estimation using multi-locational (GxE) interaction. DBB = dotted brown/black eye, LBLBE = light brown/light brown eye.

Line Name	Country of Origin	Dataset	Provided by	Growing Season	Growing Location
100SB16ANAM-C-A-16-4	Namibia	SC and SI	CFF	2016	Malaysia
104S-1913NAM	Namibia	SC and SI	CFF	2015	Malaysia
109BWA1BWA-1	Botswana	SC and SI	CFF	2015	Malaysia
20Acc118CIV-B	Ivory Coast	SC and SI	CFF	2016	Malaysia
56Acc89MLI-C	Mali	SC and SI	CFF	2015	Malaysia
84ACC696ZMB-2	Zimbabwe	SC and SI	CFF	2015	Malaysia
99SB4-2NAM-A	Namibia	SC and SI	CFF	2015	Malaysia
60ACC32NGA-A	Nigeria	SC and SI	CFF	2015	Malaysia
1-76ACC390SDN-CA11	Sudan	SC and SI	CFF	2016	Malaysia
91UNISRSWA-B	Swaziland	SC and SI	CFF	2015	Malaysia
92AHM968NAM-C	Namibia	SC and SI	CFF	2015	Malaysia
Ankpa-4	Nigeria	SC only	UoN	2016	UK
BC12105	Indonesia	SC only	CFF	2015	Malaysia
BC31107	Indonesia	SC and SI	CFF	2017	Malaysia
BCGC12107	Indonesia	SC only	CFF	2015	Malaysia
BCGC23107	Indonesia	SC and SI	CFF	2015	Malaysia
BCL33107	Indonesia	SC only	CFF	2017	Malaysia
BH16107	Indonesia	SC and SI	CFF	2017	Malaysia
BH17107	Indonesia	SC only	CFF	2015	Malaysia
BD	Indonesia	SC and SI	CFF	2015	Malaysia
Bogor	Indonesia	SC only	CFF	2017	Malaysia
Burkina *	Burkina Faso	SC and SI	CFF	2015	Malaysia
Cikur 2.1_CFF_2016	Indonesia	SC and SI	CFF	2016	Malaysia
Cikur 2.3	Indonesia	SC only	CFF	2015	Malaysia
Cikur 3.3	Indonesia	SC only	CFF	2015	Malaysia
Cream Grey Eye-49	Ghana	SC and SI	CFF	2016	Malaysia
Cream Light Brown Eye	Ghana	SC and SI	CFF	2015	Malaysia
Cream with black splash	Ghana	SC only	CFF	2015	Malaysia
DipC_CFF **	Botswana	SC and SI	CFF	2016	Malaysia
DipC_UoN **	Botswana	SC only	UoN	2016	UK
DodR_CFF **	Tanzania	SC and SI	CFF	2015	Malaysia
DodR_UoN **	Tanzania	SC only	UoN	2016	UK
DBB	Ghana	SC and SI	CFF	2015	Malaysia
Exsokoto *	Nigeria	SC only	CFF	2016	Malaysia
Exsokoto-39	Nigeria	SC and SI	CFF	2017	Malaysia
Exsokoto-177	Nigeria	SC only	CFF	2017	Malaysia
Exsokoto-183	Nigeria	SC only	CFF	2017	Malaysia
GC11105	Indonesia	SC only	CFF	2015	Malaysia
GC32105	Indonesia	SC only	CFF	2015	Malaysia
GC35107	Indonesia	SC only	CFF	2015	Malaysia
GCL13105	Indonesia	SC only	CFF	2015	Malaysia
Getso_UoN	Indonesia	SC only	UoN	2016	UK
GH17105	Indonesia	SC only	CFF	2015	Malaysia
GH21105	Indonesia	SC only	CFF	2015	Malaysia
GH36107	Indonesia	SC only	CFF	2015	Malaysia
GH37107	Indonesia	SC and SI	CFF	2015	Malaysia
GHC36105	Indonesia	SC and SI	CFF	2015	Malaysia
Gresik_CFF **	Indonesia	SC only	CFF	2016	Malaysia
Gresik_UON **	Indonesia	SC only	UoN	2016	UK
GOBRAS2.2	Indonesia	SC only	CFF	2015	Malaysia
IITA 686_CFF **/*	Nigeria	SC and SI	CFF	2016	Malaysia
IITA-686_UoN **	Nigeria	SC only	UoN	2016	UK
IPB Bam-1	Indonesia	SC and SI	CFF	2015	Malaysia
IPB Bam-2	Indonesia	SC and SI	CFF	2015	Malaysia
IPB Bam-5	Indonesia	SC only	CFF	2015	Malaysia
IPB Bam-6_CFF2016	Indonesia	SC and SI	CFF	2016	Malaysia
IPB Bam-10	Indonesia	SC only	CFF	2015	Malaysia
Kaaro	Nigeria	SC only	CFF	2016	Malaysia
Kaaro-4 *	Nigeria	SC only	CFF	2017	Malaysia
Kaaro-66	Nigeria	SC and SI	CFF	2017	Malaysia
Kaaro-74 *	Nigeria	SC only	CFF	2017	Malaysia
Kano2	Nigeria	SC only	UoN	2016	UK
Kano	Nigeria	SC only	UoN	2016	UK
LunT	Sierra Leone	SC and SI	CFF	2017	Malaysia
LBLBE	Ghana	SC and SI	CFF	2016	Malaysia
Mottled Black_2016	Ghana	SC and SI	CFF	2016	Malaysia
Nav4-13	Ghana	SC and SI	CFF	2017	Malaysia
Rajap 3.2	Indonesia	SC and SI	CFF	2015	Malaysia
S19-3_CFF **	Namibia	SC and SI	CFF	2017	Malaysia
S19-3_UoN **	Namibia	SC only	UoN	2016	UK
Situraja2	Indonesia	SC only	CFF	2016	Malaysia
Songkhla-1 *	Thailand	SC and SI	CFF	2016	Malaysia
Songkhla-1-38	Thailand	SC only	CFF	2017	Malaysia
Songkhla-1-72	Thailand	SC only	CFF	2017	Malaysia
Songkhla-1-90	Thailand	SC only	CFF	2017	Malaysia
Sukuraja-2	Indonesia	SC only	CFF	2015	Malaysia
Thung yang dang	Thailand	SC only	CFF	2015	Malaysia
Tiga Nicaru	Nigeria	SC only	UoN	2016	UK
TVSU 738	Zambia	SC only	CFF	2015	Malaysia
TVSU 89	Mali	SC and SI	CFF	2015	Malaysia
TVSu 880	Zambia	SC only	AGG	2017	Australia
TVSu 879	Zambia	SC only	AGG	2017	Australia
TVSu 702	Zambia	SC only	AGG	2017	Australia
TVSU 334	Nigeria	SC only	AGG	2017	Australia
TVSU 922	Zambia	SC only	AGG	2017	Australia
TVSU 924	Zambia	SC only	AGG	2017	Australia
TVSu 323	Nigeria	SC only	AGG	2017	Australia
TVSu 1023	Zimbabwe	SC only	AGG	2017	Australia
TVSu 1033	Zimbabwe	SC only	AGG	2017	Australia
TVSU 1231	Nigeria	SC only	AGG	2017	Australia
TVSu 702-2018	Zambia	SC only	AGG	2018	Australia
TVSu 879-2018	Zambia	SC only	AGG	2018	Australia
TVSu 880-2018	Zambia	SC only	AGG	2018	Australia
Uniswa Red	Swaziland	SC and SI	CFF	2017	Malaysia
Uniswa Red_AB_CFF **	Swaziland	SC only	CFF	2016	Malaysia
Uniswa Red-Red_UoN **	Swaziland	SC only	UoN	2016	UK
Uniswa Red-Greeen_UoN	Swaziland	SC only	UoN	2016	UK
URUG2(1)	Indonesia	SC only	CFF	2015	Malaysia
Zebra Cream-8	Ghana	SC only	CFF	2016	Malaysia
Zebra Cream-10 (KM10)	Ghana	SC and SI	CFF	2016	Malaysia

**Table 2:** List of soybean and cowpea lines used as comparator species for de novo analysis of seed proximate composition. Lines were provided by Australian Grains Genebank (AGG; Australia) and Prof. Terry Rose (TR) from Southern Cross University, Australia.

Species	Line Name	Provided by	Accession Number	Growing Season	Growing Location
Soybean	Williams82	AGG	AGG100180SOYB1 ^1^	2017	Australia
Soybean	Stuart	AGG	AGG323309SOYB1 ^1^	2017	Australia
Soybean	Essex	AGG	AGG104447SOYB1 ^1^	2017	Australia
Soybean	Peking	AGG	AGG65879SOYB4 ^1^	2017	Australia
Soybean	Asgrow	TR	N/A	2017	Australia
Soybean	Richmond	TR	N/A	2017	Australia
Soybean	Hayman	TR	N/A	2017	Australia
Cowpea	IT84S-2246-4	AGG	AGG306477COWP2 ^1^	2017	Australia
Cowpea	Han Chui Yen	AGG	AGG306534COWP2 ^1^	2017	Australia
Cowpea	524B	AGG	AGG317984COWP2 ^1^	2017	Australia

^1^ AGG accession number as per seed packets received from AGG.

**Table 3:** List of seed composition and agronomic traits used in this study with equivalent controlled vocabularies in the Crop Dietary Nutritional Ontology (CDNOhttp://www.obofoundry.org/ontology/cdno.html, accessed on 13 December 2021) [51] and the bambara groundnut Crop Ontology (CO_366; https://cropontology.org/term/CO_366:ROOT, accessed on 13 December 2021) [52]. N/A = Not available.

Trait Name	Method	Controlled Vocabulary Terms	
		Crop Dietary Nutritional Ontology ^1^	Crop Ontology ^2^
Moisture	AOAC 925.09	CDNO:0200002	CO_366:0000185
Ash	AOAC 942.05	CDNO:0200004	N/A
Lipid	AOAC 948.22	CDNO:0200068	CO_366:0000023
Protein	AOAC 992.23	CDNO:0200040	CO_366:0000026
Total carbohydrate	N/A	CDNO:0200005	CO_366:0000309
Fatty acid composition	AOAC996.06	CDNO:0200465	N/A
Oleic acid	AOAC996.06	CDNO:0200085	N/A
Lignoceric acid	AOAC996.06	CDNO:0200081	N/A
Linoleic acid	AOAC996.06	CDNO:0200102	N/A
Linolenic acid	AOAC996.06	CDNO:0200097	N/A
Seed number	IPGRI descriptor	N/A	CO_366:0000340
Dry seed weight	IPGRI descriptor	N/A	CO_366:0000337
Dry pod weight	IPGRI descriptor	N/A	CO_366:0000325
Single seed size	IPGRI descriptor	N/A	CO_366:0000328
Shelling percentage	IPGRI descriptor	N/A	CO_366:0000334

^1^ The Crop Dietary Nutritional Ontology (CDNO) provides structured terminologies to describe nutritional attributes of material entities that contribute to human diet. ^2^ Crop Ontology (CO) for bambara groundnut is curated by Liliana Andres and Graham King from Southern Cross University.

**Table 4:** Description of selection indices and the scenarios tested within each index in this study. Five indices and 15 scenarios were tested. Selection parameters (trait weightings and traits selected) for each scenario is indicated by corresponding SI number (1–5), and alphabets (a–e). For example, we tested four scenarios in SI3 using combinations of oleic acid and different yield traits and (3a–3d).

Selection Index	Potential End User	Scenarios Tested	Traits Selected for Scenario	Trait Weighting
Seed lipid (SI-1)	Nutritionist, food industry, plant breeder	(1a) Oleic acid	(1a) Oleic acid	3
Omega 3 and 6 fatty acids (SI-2)	Nutritionist, food industry, plant breeder	(2a) Linoleic acid and Linolenic acid	(2a) Linoleic acid Linolenic acid	3 3
Seed lipid and yield (SI-3)	Plant breeder	(3a) Oleic acid and seed weight (3b) Oleic acid and single seed size (3c) Oleic acid and seed number (3d) Oleic acid, seed number, and seed weight	(3a) Oleic acid Seed weight (3b) Oleic acid Seed size (3c) Oleic acid Seed number (3d) Oleic acid Seed number Seed weight	3 2 3 2 3 2 3 2 2
Omega 3, 6 fatty acid and yield (SI-4)	Plant breeder	(4a) Linoleic acid, linolenic acid, and seed weight (4b) Linoleic acid, linolenic acid, and single seed size (4c) Linoleic acid, Linolenic acid, and seed number (4d) Linoleic acid, linolenic acid seed number, and seed weight	(4a) Linoleic acid Linolenic acid Seed weight (4b) Linoleic acid Linolenic acid Seed size (4c) Linoleic acid Linolenic acid Seed number (4d) Linoleic acid Linolenic acid Seed number Seed weight	3 3 2 3 3 2 3 3 2 3 3 2 2
Yield (SI-5)	Farmer	(5a) Seed weight (5b) Seed number (5c) Single seed size (5d) Seed number and seed weight (5e) Seed number, seed weight, and single seed size	(5a) Seed weight (5b) Seed number (5c) Seed size (5d) Seed number Seed weight (5e) Seed number Seed weight Seed size	2 2 2 2 2 2 2 2

**Table 5:** Correlation analysis output of 40 bambara groundnut lines for the following traits: seed number, pod dry weight, seed dry weight, single seed size, shelling percent (%), seed lipid, protein, carbohydrate, and oleic, lignoceric, linoleic, and linolenic acids. Pearson’s correlation coefficient (r values) were considered significant at *p* < 0.05 for r > 0.312 (*n* = 40, d.f = 38). Significant correlations between traits are in bold. Carb = carbohydrate; DW = dry weight, SSS = single seed size.

	Controlled Vocabulary ^1^	Seed Number	Pod DW	Seed DW	SSS	Shelling %	Seed Protein	Seed Lipid	Seed Carb	Oleic Acid	Lignoceric Acid	Linoleic Acid	Linolenic Acid
Seed number	CO366:0000340	1.00											
Pod DW	CO366:0000325	**0.88**	1.00										
Seed DW	CO366:0000337	**0.94**	**0.97**	1.00									
SSS	CO366:0000328	**0.42**	**0.65**	**0.62**	1.00								
Shelling %	CO366:0000334	−0.30	**−0.49**	**−0.37**	**−0.32**	1.00							
Seed Protein	CDNO:0200040 CO366:0000026	−0.11	0.08	0.02	0.25	−0.15	1.00						
Seed Lipid	CDNO:0200068 CO366:0000023	**0.44**	**0.38**	**0.41**	0.31	−0.20	0.09	1.00					
Seed Carb	CDNO:0200005 CO366:0000309	0.00	−0.10	−0.07	−0.22	0.14	−0.91	−0.40	1.00				
Oleic acid	CDNO:0200085	**0.43**	**0.42**	**0.43**	0.35	−0.28	0.30	**0.62**	−0.43	1.00			
Lignoceric acid	CDNO:0200081	**0.48**	**0.47**	**0.51**	**0.32**	−0.26	0.18	**0.38**	−0.21	0.55	1.00		
Linoleic acid	CDNO:0200102	−0.15	−0.16	−0.18	−0.16	0.04	0.07	−0.19	−0.04	**−0.35**	−0.28	1.00	
Linolenic acid	CDNO:0200097	**−0.46**	**−0.43**	**−0.43**	**−0.30**	**0.36**	−0.16	−0.78	0.37	−0.63	−0.38	0.05	1.00

^1^ Equivalent controlled vocabulary from the Crop Dietary Nutritional Ontology (CDNO) [51] and bambara groundnut crop ontology (CO_366) [52] for each trait is included.

**Table 6:** Estimation of heritability using two methods: parent–offspring regression analysis and GxE analysis using two subsets of bambara groundnut lines as described in heritability analysis (Section 2.4) Input data for nutritional traits were mean values calculated from technical triplicate analysis, expressed in g/100 g seed dry weight (Supplementary Table S3). Input data for agronomic traits were mean values for the bambara groundnut line calculated from all replicates for the line (Supplementary Table S4). SSS = Single seed size; Carb = carbohydrate; DW = dry weight.

	Seed Number	Pod DW	Seed DW	SSS	Shelling %	Protein	Lipid	Carb	Linoleic Acid	Oleic Acid	Lignoceric Acid	Linolenic Acid
*Heritability from parent to offspring (regression)*												
Generation 1 to 2	37	20	28	67	22	27	34	35	9	67	77	44
Generation 2 to 3	41	6	12	16	7	72	45	58	36	79	62	32
*Heritability from GxE analysis*												
Heritability	n/a	n/a	n/a	n/a	n/a	77	63	17	0	0	69	81
*Average across three estimates of heritability*												
Average	26	19	13	28	10	59	47	37	15	49	69	52

**Table 7:** Summary of bambara groundnut lines with positive scores for all scenarios tested in each of the indices (SI): seed lipid (SI-1), omega 3 and 6 fatty acids (SI-2), seed lipid and yield (SI-3), omega 3 and 6 fatty acids and yield (SI-4), and yield (SI-5). Positive scores are indicated with a plus sign (+).

Bambara Groundnut Line	Geographical Origin	SI-1 Seed Lipid	SI-2 Omega 3 and 6 FA	SI-3 Seed Lipid and Yield	SI-4 Omega 3 and 6 FA and Yield	SI-5 Yield Only
IPB-Bam2	Indonesia	+	+	+	+	+
IPB-Bam1	Indonesia	+	+	+	+	+
GHC36105	Indonesia	+	+	+	+	+
99SB4-2NAM-A	Namibia	+	+	+	+	+
IPB Bam-6	Indonesia	+	+	+	+	+
BC31107	Indonesia	+	+	+	+	+
56Acc89MLI-C	Mali	+	+		+	+
109BWA1BWA-1	Botswana	+		+	+	+
DodR_CFF	Tanzania	+	+	+	+	+
BD	Indonesia	+		+		+
Cikur 2.1,	Indonesia	+		+		+
104S-1913NAM	Namibia	+		+		+
91UNISRSWA-B	Swaziland	+		+		+
BCGC23107	Indonesia	+		+		
BH16107	Indonesia	+				
92AHM968NAM-C	Namibia	+		+		+
100SB16ANMAMCA16	Namibia	+		+		
LunT	Sierra Leone			+		
Mottle Black	Ghana		+		+	
Nav4-13	Ghana		+		+	
TVSU89	Thailand		+		+	
Zebra Cream-10	Ghana		+		+	
LBLBE	Ghana		+		+	
DBB	Ghana		+		+	
IITA686_CFF_2016	Nigeria				+	+

**Table 8:** Expected percent (%) change to value of each trait value using the five selection indices. Calculation was based on 15 bambara groundnut lines with the highest score for each index. Calculation of percent expected to change as outlined in Selection index and Scenario Testing (Section 2.5) Single seed size = SSS; Carb = carbohydrate; DW = dry weight.

	Percent of Change to Trait Value from Mean Value (%)										
Selection Index	Seed Number	Pod DW	Seed DW	SSS	Shelling %	Lipid	Protein	Carb	Linoleic Acid	Oleic Acid	Linolenic Acid
1: Seed lipid	53.49	51.52	63.44	13.10	2.21	8.34	3.71	−1.84	−2.52	8.93	−7.54
2: Omega 3 and 6 fatty acid	7.45	32.73	34.52	16.00	4.89	−7.40	2.73	0.28	2.62	−5.64	19.89
3: Seed lipid and yield	64.88	67.93	74.86	12.57	−3.24	7.28	2.39	−1.29	−2.70	9.05	−9.26
4: Omega 3,6 fatty acid and yield	39.74	40.13	49.99	11.68	5.49	−2.51	0.25	0.26	0.57	−1.83	13.89
5: Yield	76.23	76.89	86.24	14.70	−3.46	5.26	−0.70	−0.21	−2.02	5.24	−4.77

## Data Availability

Not applicable.

## Supplementary Materials

The following are available online at https://www.mdpi.com/article/10.3390/foods11010086/s1, Table S1: Proximate composition of 100 bambara groundnut lines, 7 soybean lines, and 3 cowpea lines, Table S2: Fatty acid composition of 100 bambara groundnut lines, 7 soybean lines, and 3 cowpea lines, Figure S1: Principal component analysis (PCA) loading plot for seed lipid concentration (g/100g dry weight) and 8 fatty acids for the global diversity set of 100 bambara groundnut lines, Figure S2: PCA loading plot of agronomic (seed number, pod dry weight, seed dry weight, and seed size, shelling percentage) and nutritional traits (seed lipid, protein, carbohydrate, oleic acid, lignoceric acid, linoleic acid, and linolenic acid) selected for selection indices dataset of 40 bambara groundnut lines, Table S3: Scenario scores calculated from SND values presented in for all 15 scenarios tested for the 5 selection indices, Table S4: Scenario scores for the bambara groundnut lines with the highest 15 scores for scenario with each selection index.
