# Supplementary material for: Development of Selection Indices for Improvement of Seed Yield and Lipid Composition in Bambara Groundnut (Vigna subterranea (L.) Verdc.)
Source: Foods. 2021 Dec 29;11(1):86. doi: 10.3390/foods11010086 (PMC8750730; doi:10.3390/foods11010086)
Supplement: Supplementary file 1 [file foods-11-00086-s001.zip › foods-1488784-supplementary.pdf]

Table S1: Proximate composition of 100 bambara groundnut lines, seven soybean lines and three cowpea lines expressed in g/100g dry weight, except for moisture which is expressed in g/100g of seed fresh weight. Mean values ± standard deviation of technical triplicate analysis. Significance of differences between means of accessions indicated by (**) are p<0.01. Mean values and standard deviation calculated from experimental raw data values. DBB=Dotted brown/black eye, LBLBE=Light brown/light brown eye

Line ID	Line	Moisture	Ash	Crude protein	Lipid	Total carbohydrate	Metabolised Energy (kJ)
**Bambara groundnut**							
RA1700101	100SB16ANAM-C	6.36± 0.32	6.07±0.06	22.43±0.13	7.35±0.07	64.16±0.20	1673.12±0.56
RA1700102	104S-1913NAM	6.44± 0.11	4.50±0.16	19.73±0.10	8.33±0.17	67.44±0.33	1724.04±2.96
RA1700103	109BWA1BWA-1	6.16 ± 0.58	5.52±0.17	15.99±0.16	6.91±0.09	71.59±0.42	1690.52±1.68
RA1700104	20Acc118CIV-B	6.83 ± 0.52	5.94±0.10	22.67±0.21	7.82±0.12	63.57±0.31	1683.01±1.08
RA1700105	56Acc89MLI-C	5.87± 0.12	6.18±0.20	17.22±0.05	8.08±0.06	68.51±0.22	1697.35±3.34
RA1700106	84ACC696ZMB-2	6.48± 0.09	6.61±0.05	14.81±0.12	7.99±0.16	70.58±0.19	1694.42±2.50
RA1700107	99SB4-2NAM-A	6.23± 0.01	6.33±0.07	20.15±0.10	7.86±0.05	65.66±0.10	1683.41±1.58
RA1700108	60ACC32NGA-A	6.05± 0.62	4.56±0.28	14.75±0.13	6.55±0.11	74.14±0.45	1703.56±3.30
RA1700109	1-76ACC390SDN-CA11	7.63± 0.86	7.72±0.20	24.85±0.25	6.33±0.03	61.46±0.47	1620.66±3.38
RA1700110	91UNISRSWA-B	5.94± 0.22	4.01±0.10	18.67±0.19	6.95±0.05	70.37±0.10	1710.14±1.81
RA1700111	92AHM968NAM-C	7.49± 0.05	4.21±0.10	17.59±0.21	7.07±0.16	71.13±0.38	1711.80±3.10
RA1700112	Ankpa4	7.21± 0.50	5.72±0.16	24.45±0.38	5.41±0.07	64.42±0.60	1639.09±2.54
RA1700113	BC12105	6.41±0.17	5.22±0.15	22.80±0.12	7.80±0.01	64.19±0.24	1694.64±3.03
RA1700114	BC31107	5.86±0.35	5.93±0.13	21.31±0.08	6.58±0.03	66.18±0.22	1664.40±1.98
RA1700115	BCGC12107	5.99± 0.45	4.49±0.04	21.52±0.09	8.06±0.14	65.93±0.24	1714.90±1.79
RA1700116	BCGC23107	5.74± 0.13	4.64±0.09	19.49±0.16	8.14±0.05	67.72±0.19	1718.96±2.75
RA1700117	BCL33107	6.39± 0.19	5.85±0.17	21.97±0.04	6.72±0.02	65.46±0.16	1666.62±3.04
RA1700118	BH16107	7.95± 0.53	5.33±0.11	23.94±0.04	6.79±0.09	63.93±0.18	1671.84±2.08
RA1700119	BH17107	6.36± 0.20	5.61±0.28	21.34±0.06	7.03±0.07	66.02±0.38	1677.90±3.81
RA1700120	BD	6.18± 0.23	4.83±0.34	18.99±0.07	8.76±0.06	67.42±0.29	1728.20±5.47
RA1700121	Bogor	4.64± 0.04	3.97±0.08	24.71±0.17	5.89±0.50	65.43±0.58	1676.75±8.33
RA1700122	Burkina	6.30± 0.04	4.17±0.05	20.17±0.11	6.27±0.12	69.39±0.25	1691.68±1.31

Line ID	Line name	Moisture	Ash	Crude protein	Lipid	Total carbohydrate	Metabolised Energy (kJ)
RA1700123	Cikur2.1	7.13± 0.34	5.97±0.21	23.51±0.23	7.41±0.09	63.10±0.10	1673.18±4.75
RA1700124	Cikur2.3	5.64± 0.51	5.92±0.12	17.44±0.08	7.78±0.09	68.86±0.05	1695.72±3.39
RA1700125	Cikur3.3	6.85± 0.33	5.20±0.49	20.53±0.25	7.85±0.09	66.41±0.52	1701.56±7.07
RA1700126	Cream Grey Eye-49	6.32± 0.36	5.64±0.26	16.50±0.11	6.52±0.08	71.35±0.28	1680.18±5.70
RA1700127	Cream Light Brown Eye	5.65± 0.75	6.14±0.05	18.88±0.21	6.22±0.05	68.76±0.22	1660.43±2.16
RA1700128	Cream with black splash	6.26± 0.60	6.45±0.18	17.42±0.13	5.37±0.01	70.75±0.28	1643.32±3.45
RA1700129	DipC_CFF	8.14± 0.10	4.36±0.10	20.41±0.02	5.87±0.11	69.36±0.06	1680.59±3.51
RA1700130	DipC_UoN	8.19± 0.24	5.02±0.23	25.23±0.11	6.96±0.07	62.79±0.19	1676.87±2.40
RA1700131	DodR_CFF	7.08± 0.22	6.11±0.05	18.14±0.12	6.80±0.13	68.95±0.21	1673.26±2.92
RA1700132	DodR_UoN	7.80± 0.14	5.66±0.18	21.11±0.16	7.26±0.05	65.97±0.25	1681.73±3.06
RA1700133	DBB	6.38± 0.37	5.76±0.16	17.05±0.06	6.35±0.07	70.85±0.12	1673.73±3.88
RA1700134	Exsokoto	7.21± 0.56	5.95±0.07	21.96±0.16	6.84±0.14	65.25±0.28	1667.12±2.95
RA1700135	Exsokoto-39	6.57± 0.09	5.46±0.19	21.63±0.25	7.30±0.02	65.62±0.08	1684.51±2.95
RA1700136	Exsokoto-177	6.31± 0.37	5.69±0.03	21.79±0.14	6.06±0.28	66.46±0.42	1657.79±4.40
RA1700137	Exsokoto-183	5.07± 0.06	5.42±0.60	23.81±0.25	6.35±0.04	64.42±0.45	1662.65±10.34
RA1700138	GC11105	4.71± 0.35	4.39±0.13	23.18±0.11	8.36±0.06	64.07±0.24	1717.92±2.55
RA1700139	GC32105	4.67± 0.58	4.58±0.21	19.36±0.06	8.05±0.07	68.01±0.30	1718.62±3.43
RA1700140	GC35107	6.39± 0.08	4.03±0.08	24.33±0.12	7.79±0.18	63.85±0.25	1710.84±4.10
RA1700141	GCL13105	6.93± 0.12	3.82±0.23	22.72±0.05	8.24±0.10	65.22±0.37	1726.61±2.54
RA1700142	Getso	6.61±0.05	5.74±0.20	27.15±0.55	5.73±0.15	61.38±0.52	1637.62±4.98
RA1700143	GH17105	7.01± 0.23	3.80±0.15	21.71±0.13	8.60±0.29	65.90±0.55	1735.90±2.80
RA1700144	GH21105	7.38± 0.23	4.30±0.30	22.47±0.06	7.04±0.11	66.18±0.37	1697.39±5.53
RA1700145	GH36107	6.89± 0.01	5.25±0.29	21.61±0.14	7.57±0.21	65.57±0.45	1693.06±5.32
RA1700146	GH37107	5.74± 0.21	3.92±0.15	21.59±0.01	8.71±0.06	65.78±0.09	1736.28±3.58
RA1700147	GHC36105	7.11± 0.08	4.92±0.13	20.63±0.12	6.56±0.17	67.89±0.30	1682.76±3.87
RA1700148	Gresik_CFF_2016	6.74± 0.14	4.33±0.05	22.05±0.13	5.21±0.22	68.41±0.32	1665.02±3.62
RA1700149	Gresik_UoN	8.52± 0.32	6.21±0.20	26.33±0.13	6.13±0.02	61.33±0.32	1639.03±3.79
RA1700150	GOBRAS2.2	6.43± 0.14	3.75±0.09	17.81±0.11	7.72±0.10	70.71±0.28	1730.62±1.33
RA1700151	IITA 686_CFF	6.40± 0.15	5.36±0.07	16.76±0.09	6.50±0.04	71.38±0.06	1684.07±1.02
RA1700152	IITA-686_UoN	8.22± 0.09	5.76±0.22	19.63±0.15	6.92±0.12	67.69±0.43	1677.56±3.52

Line ID	Line name	Moisture	Ash	Crude protein	Lipid	Total carbohydrate	Metabolised Energy (kJ)
RA1700153	IPB Bam-1	5.94± 0.15	4.06±0.14	23.08±0.03	7.71±0.05	65.15±0.09	1712.06±2.82
RA1700154	IPB Bam-2	5.83± 0.12	4.55±0.20	20.80±0.19	7.46±0.09	67.20±0.24	1704.97±1.66
RA1700155	IPB Bam-5	6.01± 0.02	4.42±0.19	21.48±0.13	7.93±0.07	66.16±0.21	1713.95±2.37
RA1700156	IPB Bam-6	6.18± 0.04	5.00±0.15	25.18±0.12	6.35±0.04	63.47±0.17	1666.21±2.79
RA1700157	IPB Bam-10	5.67± 0.20	4.49±0.03	23.90±0.11	7.74±0.08	63.88±0.13	1703.10±2.05
RA1700158	Kaaro	5.02± 0.15	4.72±0.05	21.56±0.04	5.44±0.03	68.27±0.03	1663.76±1.06
RA1700159	Kaaro-4	5.91± 0.05	5.32±0.08	22.85±0.08	5.46±0.05	66.36±0.13	1650.73±0.61
RA1700160	Kaaro-66	5.49± 0.27	5.05±0.03	20.61±0.04	6.06±0.02	68.28±0.04	1671.55±0.99
RA1700161	Kaaro-74	5.16± 0.06	5.24±0.10	19.68±0.17	5.98±0.05	69.10±0.22	1669.37±2.87
RA1700162	Kano2	8.11± 0.32	5.77±0.22	22.56±0.37	6.60±0.11	65.07±0.39	1664.22±5.66
RA1700163	Kano3	8.41± 0.52	5.56±0.30	24.98±0.51	6.49±0.01	62.97±0.31	1659.87±4.20
RA1700164	LunT	8.05± 0.25	4.14±0.20	17.90±0.15	5.81±0.17	72.15±0.23	1689.45±4.34
RA1700165	LBLBE	5.52± 0.26	4.56±0.02	17.11±0.12	5.94±0.03	72.39±0.08	1686.60±0.71
RA1700166	Mottled Black	6.74± 0.51	4.30±0.09	18.65±0.04	4.24±0.12	72.81±0.14	1656.55±2.92
RA1700167	Nav4-13	6.52± 0.33	5.02±0.25	16.97±0.11	6.26±0.03	71.76±0.25	1684.89±4.61
RA1700168	Rajap 3.2	7.17± 0.37	4.17±0.13	22.42±0.06	6.92±0.11	66.49±0.02	1697.62±3.85
RA1700169	S19-3_CFF	5.47± 0.30	5.15±0.01	20.63±0.12	6.61±0.08	67.61±0.20	1679.83±0.99
RA1700170	S19-3_UoN	8.46± 0.35	5.84±0.24	28.88±0.01	6.39±0.03	58.89±0.26	1643.48±3.87
RA1700171	Situraja2	7.39± 0.18	4.66±0.31	22.16±0.09	6.90±0.08	66.28±0.31	1689.55±3.64
RA1700172	Songkhla-1	5.18± 0.04	5.09±0.09	18.90±0.03	7.37±0.06	68.65±0.04	1698.84±2.49
RA1700173	Songkhla-1-38	5.67± 0.04	5.59±0.12	17.87±0.07	7.14±0.02	69.41±0.16	1688.81±1.85
RA1700174	Songkhla-1-72	5.88± 0.09	5.49±0.05	18.22±0.09	6.99±0.18	69.31±0.29	1686.98±2.72
RA1700175	Songkhla-1-90	5.68± 0.17	5.21±0.07	17.43±0.05	6.21±0.47	71.15±0.42	1679.64±8.67
RA1700176	Sukuraja-2	3.36± 0.13	3.67±0.04	14.57±0.12	6.85±0.20	74.91±0.10	1724.33±4.18
RA1700177	Thung yang dang	3.77± 0.20	4.16±0.14	18.23±0.11	7.21±0.22	70.41±0.45	1713.45±2.42
RA1700178	Tiga Nicaru	7.69± 0.10	5.45±0.03	22.10±0.22	6.54±0.09	65.92±0.22	1669.93±2.43
RA1700179	TVSU738	6.44± 0.20	5.02±0.21	20.13±0.07	5.77±0.08	69.08±0.23	1668.31±1.89
RA1700180	TVSU89	4.02± 0.03	4.71±0.03	21.92±0.04	6.32±0.14	67.04±0.12	1678.93±2.34
RA1700181	TVSu880	4.52± 0.27	4.82±0.03	18.98±0.12	6.88±0.09	69.32±0.19	1694.49±1.32

Line ID	Line name	Moisture	Ash	Crude protein	Lipid	Total carbohydrate	Metabolised Energy (kJ)
RA1700182	TVSu879	5.02± 0.25	4.78±0.11	20.97±0.17	6.73±0.07	67.52±0.92	1687.41±2.45
RA1700183	TVSu702	4.54± 0.14	4.36±0.03	18.29±0.04	8.30±0.21	69.05±0.15	1729.47±3.55
RA1700184	TVSU 334	4.46± 0.19	4.71±0.11	21.57±0.16	6.58±0.10	67.14±0.94	1684.39±2.21
RA1700185	TVSU 922	7.29± 0.17	4.88±0.15	20.47±0.19	7.43±0.02	67.22±0.30	1699.67±2.68
RA1700186	TVSU 924	4.94± 0.07	5.13±0.03	20.91±0.40	7.50±0.07	66.46±0.27	1695.48±0.50
RA1700187	TVSu 323	6.37± 0.18	5.59±0.11	25.03±0.25	6.01±0.04	63.37±0.97	1650.57±1.72
RA1700188	TVSU1023	6.24± 0.20	5.23±0.22	21.02±0.35	7.47±0.07	66.28±0.73	1692.88±3.34
RA1700189	TVSU1033	4.77± 0.22	5.13±0.12	22.99±0.18	6.27±0.05	65.61±0.12	1668.25±1.28
RA1700190	TVSU 1231	5.15± 0.37	4.37±0.08	21.15±0.08	4.97±0.05	69.50±0.08	1662.25±1.05
RA1700191	TVSU702-2018	5.14± 0.29	5.93±0.25	19.94±0.06	6.51±0.15	67.62±0.30	1666.40±4.25
RA1700192	TVSU879-2018	5.69± 0.01	5.82±0.02	18.58±0.15	5.95±0.09	69.65±0.09	1661.74±2.27
RA1700193	TVSU880-2018	5.14± 0.05	5.80±0.05	20.71±0.17	6.16±0.53	67.33±0.72	1660.61±8.56
RA1700194	Uniswa Red	5.25± 0.44	4.30±0.24	18.65±0.23	6.84±0.03	70.22±0.46	1703.33±4.66
RA1700195	Uniswa Red _AB_CFF	7.40± 0.78	4.49±0.15	16.92±0.19	6.28±0.07	72.31±0.15	1694.45±2.99
RA1700196	Uniswa Red_Red_UoN	8.57± 0.10	5.21±0.05	22.26±0.08	7.53±0.11	65.00±0.16	1691.36±1.75
RA1700197	Uniswa Red_Green_UoN	8.17± 0.11	6.25±0.17	26.54±0.24	5.90±0.07	61.30±0.45	1633.71±2.99
RA1700198	URUG2(1)	4.30± 0.13	3.77±0.13	23.27±0.06	7.74±0.21	65.22±0.32	1716.91±4.07
RA1700199	Zebra Cream-8	4.16± 0.09	4.16±0.17	17.02±0.08	5.64±0.17	73.17±0.29	1688.23±4.56
RA1700200	Zebra Cream-10	3.62±0.18	3.93±0.11	22.76±0.11	5.85±0.02	67.46±0.20	1681.61±1.60
Overall Min		3.36	3.68	14.57	4.24	58.65	1618.11
Overall Max		8.57	7.35	28.88	8.76	75.00	1738.59
Overall Mean		6.22	5.07	20.81	6.84	67.28	1684.83
Overall standard deviation		1.20	0.77	2.84	0.90	3.08	24.40
Significance of line differences		**	**	**	**	**	**
**Soybean**							
RA1700201	Williams82	4.83±0.05	7.80±0.13	43.48±0.36	15.88±0.12	32.84±0.58	1744.40±1.67
RA1700202	Stuart	3.80±0.13	6.42±0.15	46.02±0.14	17.39±0.19	30.17±0.36	1788.71±0.99
RA1700203	Essex	4.19±0.02	7.54±0.14	42.03±0.19	19.74±0.13	30.7±0.36	1822.04±3.48
RA1700204	Peking	4.00±0.09	7.74±0.53	49.91±0.41	15.52±0.24	26.83±0.68	1722.93±8.59
RA1700205	Asgrow	4.35±0.22	6.20±0.15	44.63±0.25	20.33±0.23	28.85±0.53	1848.98±4.01

RA1700206	Richmond	4.99±0.01	5.92±0.07	43.37±0.31	19.53±0.12	31.18±0.35	1842.55±1.24
RA1700207	Hayman	4.38±0.03	6.98±0.02	40.90±0.17	21.23±0.32	30.89±0.49	1861.23±5.35
Overall Min	3.65	5.85		40.71	15.26	26.07	1716.64
Overall Max	5.00	8.16		50.25	21.45	33.47	1865.14
Overall Mean	4.36	6.94		44.33	18.51	30.21	1804.41
Overall standard deviation	0.42	0.76		2.82	2.14	1.86	51.32
Significance of line differences		**	**	**	**	**	**
**Cowpea**							
RA1700215	IT84S-2246-4	6.46±0.42	5.19±0.04	25.52±0.16	1.54±0.17	62.83±0.19	1575.79±2.66
RA1700216	Han Chui Yen	4.91±0.44	5.83±0.09	28.39±0.11	1.66±0.06	62.36±0.06	1559.81±2.50
RA1700217	524B	5.08±0.08	5.42±0.02	24.08±0.10	1.54±0.21	65.18±0.28	1575.37±3.85
Overall Min		4.91	5.15	24.02	1.31	62.29	1557.04
Overall Max		6.46	5.94	28.51	1.73	65.46	1578.86
Overall standard deviation		0.85	0.29	1.91	0.15	1.32	8.32
Significance of line differences		**	**	**	*	**	**

Table S2: Fatty acid composition of 100 bambara groundnut (*V. subterranea*) lines, seven soybean lines and three cowpea lines expressed as % of total fatty acid. Mean values ± standard deviation of technical triplicate analysis. Significance of differences between means of lines indicated by (**) are p<0.01. Mean values and standard deviation calculated from raw data values. DBB=Dotted brown/black eye, LBLBE=Light brown/light brown eye.

Line ID	Line	Linoleic Acid	Oleic Acid	Palmitic acid	Stearic acid	Behenic Acid	Arachidic Acid	Lignoceric acid	α-Linolenic Acid
RA1700101	100SB16ANAM-C	35.06±0.12	25.32±0.62	16.57±0.07	6.57±0.045	5.98±0.054	2.10±0.01	2.96±0.04	2.40±0.02
RA1700102	104S-1913NAM	34.60±0.05	23.19±0.03	17.22±0.02	8.08±0.02	6.39±0.039	2.50±0.01	3.05±0.03	1.90±0.01
RA1700103	109BWA1BWA-1	36.43±0.02	20.76±0.03	19.15±0.05	6.72±0.01	5.71±0.018	2.24±0.00	3.10±0.01	2.81±0.00
RA1700104	20Acc118CIV-B	35.80±0.02	23.95±0.01	16.51±0.01	7.19±0.00	6.02±0.012	2.31±0.01	2.80±0.02	2.17±0.00
RA1700105	56Acc89MLI-C	36.96±0.03	24.16±0.09	17.18±0.03	8.29±0.01	4.42±0.003	2.48±0.01	1.44±0.00	2.34±0.05
RA1700106	84ACC696ZMB-2	37.86±0.10	20.51±0.10	16.76±0.03	7.03±0.02	6.85±0.057	2.35±0.01	3.50±0.03	2.11±0.01
RA1700107	99SB4-2NAM-A	34.22±0.05	24.38±0.04	16.64±0.03	6.71±0.02	6.85±0.022	2.19±0.00	3.60±0.02	2.04±0.02
RA1700108	60ACC32NGA-A	38.46±0.49	17.52±0.28	18.66±0.05	11.40±0.04	4.95±0.052	3.28±0.02	1.49±0.03	2.41±0.14
RA1700109	1-76ACC390SDN-CA11	39.27±0.09	20.53±0.01	18.74±0.06	8.43±0.03	3.89±0.014	2.35±0.09	1.49±0.01	2.28±0.13

RA1700110	91UNISRSWA-B	36.69±0.21	22.68±0.12	17.12±0.01	6.33±0.03	6.22±0.001	2.17±0.01	3.21±0.01	2.26±0.07
RA1700111	92AHM968NAM-C	35.98±0.05	21.52±0.11	16.74±0.09	6.55±0.05	7.36±0.060	2.20±0.01	3.70±0.10	2.28±0.10
RA1700112	Ankpa4	45.64±0.15	17.00±0.06	19.51±0.05	2.44±0.01	3.42±0.024	0.90±0.00	2.80±0.01	4.54±0.01
RA1700113	BC12105	35.27±0.01	24.99±0.49	17.97±0.02	6.98±0.00	5.28±0.004	2.13±0.00	2.57±0.02	1.96±0.01
RA1700114	BC31107	36.90±0.01	21.48±0.11	20.21±0.02	6.39±0.01	4.81±0.012	2.11±0.00	2.29±0.01	2.93±0.00
RA1700115	BCGC12107	33.00±0.02	28.32±0.56	16.65±0.02	6.97±0.01	5.50±0.011	2.12±0.01	2.68±0.01	1.68±0.00
RA1700116	BCGC23107	34.36±0.01	26.49±0.49	17.63±0.05	7.15±0.01	5.19±0.016	2.18±0.01	2.40±0.00	1.85±0.02
RA1700117	BCL33107	37.00±0.03	21.85±0.05	19.96±0.06	6.60±0.01	4.66±0.010	2.16±0.01	1.78±0.50	2.87±0.00
RA1700118	BH16107	36.47±0.04	23.33±0.26	19.15±0.03	6.47±0.01	5.08±0.013	2.13±0.01	2.33±0.02	2.77±0.00
RA1700119	BH17107	35.52±0.15	25.15±0.41	17.545±0.15	6.47±0.04	5.78±0.031	2.10±0.01	3.09±0.01	2.06±0.01
RA1700120	BD	34.63±0.05	24.88±0.38	17.67±0.14	6.30±0.04	6.32±0.13	2.22±0.21	3.53±0.39	1.87±0.22
RA1700121	Bogor	38.65±0.04	18.08±0.04	20.64±0.05	6.31±0.02	4.93±0.02	2.19±0.01	2.41±0.01	3.77±0.00
RA1700122	Burkina	41.55±0.22	17.57±0.09	18.89±0.05	8.03±0.04	4.47±0.03	2.51±0.02	1.23±0.01	2.77±0.01
RA1700123	Cikur 2.1	35.11±0.11	26.15±0.61	17.71±0.08	5.56±0.02	5.67±0.08	1.87±0.04	3.34±0.11	1.99±0.02
RA1700124	Cikur 2.3	36.32±0.21	26.90±0.18	17.32±0.02	6.53±0.03	4.42±0.05	2.06±0.01	2.42±0.03	2.06±0.10
RA1700125	Cikur 3.3	35.17±0.05	27.08±0.03	17.33±0.01	6.40±0.01	5.34±0.01	1.97±0.00	2.84±0.05	1.75±0.01
RA1700126	Cream Grey Eye-49	35.51±0.22	23.33±0.37	16.19±0.07	10.86±0.07	5.34±0.03	3.19±0.02	1.69±0.01	2.51±0.01
RA1700127	Cream Light Brown Eye	39.64±0.11	18.70±0.21	19.27±0.03	8.12±0.03	5.22±0.02	2.53±0.02	1.69±0.02	2.79±0.01
RA1700128	Cream with black splash	39.08±0.21	18.32±0.12	18.24±0.11	9.21±0.03	4.93±0.02	2.75±0.02	2.03±0.01	3.44±0.02
RA1700129	DipC_CFF	36.79±0.05	21.98±0.03	17.02±0.02	6.65±0.06	6.92±0.04	2.24±0.01	2.53±0.02	2.50±0.02
RA1700130	DipC_UoN	39.34±0.29	18.78±0.23	20.37±0.13	4.39±0.00	5.28±0.02	1.57±0.01	2.28±0.00	2.94±0.01
RA1700131	DodR_CFF	35.26±0.15	20.93±0.33	17.46±0.06	9.01±0.10	7.11±0.050	2.91±0.02	2.78±0.02	2.55±0.01
RA1700132	DodR_UoN	36.44±0.86	18.83±0.69	18.84±0.31	6.47±0.14	6.44±0.08	2.15±0.03	3.33±0.08	2.33±0.04
RA1700133	DBB	42.19±0.01	17.85±0.24	19.98±0.03	7.59±0.03	4.12±0.01	2.29±0.01	1.13±0.01	2.91±0.00
RA1700134	Exsokoto	40.65±0.06	15.84±0.04	21.31±0.00	8.71±0.05	4.67±0.01	2.82±0.01	1.64±0.00	2.43±0.01
RA1700135	Exsokoto-39	40.88±0.17	16.45±0.10	20.17±0.08	9.93±0.05	4.25±0.02	2.96±0.01	1.30±0.15	2.16±0.02
RA1700136	Exsokoto-177	40.40±0.13	14.85±0.17	21.54±0.10	8.46±0.04	5.17±0.02	2.89±0.02	1.90±0.01	2.94±0.01
RA1700137	Exsokoto-183	40.92±0.55	16.51±0.39	19.52±0.30	8.29±0.10	4.85±0.46	2.71±0.02	2.13±0.51	2.53±0.05
RA1700138	GC11105	32.06±0.07	27.14±0.02	16.68±0.03	6.20±0.02	6.21±0.01	1.97±0.01	4.10±0.03	1.64±0.00
RA1700139	GC32105	33.31±0.42	24.73±0.04	17.15±0.06	7.59±0.10	6.27±0.10	2.40±0.04	3.35±0.08	1.87±0.05
RA1700140	GC35107	33.31±0.18	29.21±0.08	17.06±0.05	6.40±0.01	5.06±0.02	1.88±0.03	2.76±0.06	1.59±0.02

RA1700141	GCL13105	33.55±0.01	25.18±0.03	16.78±0.03	6.65±0.02	6.19±0.01	2.12±0.01	4.07±0.02	1.79±0.00
RA1700142	Getso	45.23±0.00	14.62±0.00	23.27±0.01	4.35±0.00	3.49±0.00	1.46±0.00	1.65±0.00	3.37±0.00
RA1700143	GH17105	32.89±0.40	26.36±0.12	16.52±0.22	6.82±0.14	5.91±0.17	2.06±0.02	3.79±0.20	1.71±0.02
RA1700144	GH21105	33.79±0.27	27.78±0.15	17.29±0.09	6.56±0.05	5.39±0.02	2.35±0.27	2.64±0.05	1.82±0.02
RA1700145	GH36107	32.85±0.14	24.89±0.12	17.19±0.14	6.48±0.05	6.50±0.19	2.15±0.01	4.24±0.20	1.89±0.01
RA1700146	GH37107	32.06±0.12	26.69±0.07	16.51±0.07	6.87±0.02	6.37±0.12	2.14±0.01	3.99±0.14	1.56±0.01
RA1700147	GHC36105	36.86±0.15	20.47±0.03	19.42±0.10	6.68±0.03	5.34±0.12	2.27±0.01	3.03±0.10	3.08±0.03
RA1700148	Gresik_CFF_2016	36.55±0.19	21.24±0.88	18.79±0.25	6.48±0.14	5.42±0.09	2.17±0.05	3.13±0.06	3.23±0.32
RA1700149	Gresik_UoN	38.16±0.04	20.30±0.02	21.92±0.03	3.41±0.01	4.54±0.02	1.25±0.00	3.08±0.02	3.48±0.00
RA1700150	GOBRAS2.2	34.89±0.12	23.91±0.03	16.08±0.09	6.15±0.01	6.76±0.11	2.10±0.01	4.50±0.15	1.79±0.02
RA1700151	IITA686_CFF	36.68±0.24	18.59±0.09	16.97±0.16	7.88±0.09	6.78±0.19	2.56±0.03	4.27±0.22	3.04±0.02
RA1700152	IITA686_UoN	40.40±0.24	17.89±0.12	19.32±0.13	3.79±0.04	4.50±0.03	1.33±0.01	3.20±0.06	3.29±0.01
RA1700153	IPB Bam-1	33.66±0.18	25.70±0.13	15.96±0.11	5.75±0.02	6.44±0.16	1.94±0.01	4.65±0.22	1.69±0.01
RA1700154	IPB Bam-2	33.78±0.09	25.31±0.07	16.13±0.04	5.85±0.02	6.51±0.08	1.98±0.00	4.11±0.01	1.86±0.11
RA1700155	IPB Bam-5	33.66±0.67	25.30±0.19	16.50±0.19	6.39±0.08	6.53±0.07	2.12±0.04	4.11±0.01	1.58±0.09
RA1700156	IPB Bam-6	34.71±0.38	22.55±0.27	18.34±0.45	5.57±0.58	5.89±0.51	1.89±0.09	4.73±0.04	2.56±0.19
RA1700157	IPB Bam-10	34.02±0.11	24.81±0.08	16.21±0.07	6.15±0.02	6.50±0.11	2.05±0.01	4.56±0.09	1.77±0.01
RA1700158	Kaaro-x	40.47±0.09	17.72±0.02	18.73±0.04	8.44±0.02	4.77±0.03	2.53±0.02	1.92±0.01	3.21±0.01
RA1700159	Kaaro-4	41.29±0.59	16.50±0.17	18.98±0.09	8.35±0.10	4.99±0.10	2.52±0.05	2.21±0.07	2.91±0.04
RA1700160	Kaaro-66	42.23±0.02	16.68±0.01	17.46±0.01	8.95±0.01	5.34±0.16	2.68±0.00	1.65±0.00	2.63±0.01
RA1700161	Kaaro-74	41.88±0.19	16.40±0.02	17.71±0.02	9.11±0.02	5.31±0.05	2.75±0.02	1.63±0.01	2.66±0.01
RA1700162	Kano2	42.41±0.06	15.35±0.09	23.52±0.03	5.21±0.01	3.91±0.03	1.66±0.01	1.75±0.01	3.35±0.00
RA1700163	Kano3	42.56±0.10	20.97±0.05	20.42±0.05	4.25±0.00	3.17±0.01	1.33±0.00	1.10±0.00	3.09±0.01
RA1700164	LunT	36.68±0.15	23.53±0.07	16.55±0.03	7.92±0.02	5.45±0.04	2.41±0.01	2.74±0.02	1.77±0.01
RA1700165	LBLBE	37.75±0.03	17.23±0.02	19.71±0.02	8.90±0.01	5.26±0.04	2.68±0.01	2.09±.0.02	3.16±0.00
RA1700166	Mottled Black	37.19±0.08	19.09±0.16	18.81±0.15	8.54±0.03	4.33±0.01	2.48±0.00	2.48±0.02	4.25±0.02
RA1700167	Nav4-13 (NAV0983)	39.36±0.02	16.37±0.01	19.51±0.05	8.71±0.00	5.65±0.02	2.72±0.01	1.57±0.00	3.24±0.00
RA1700168	Rajap 3.2	38.17±0.16	23.42±0.01	17.73±0.08	6.44±0.37	4.14±0.02	2.09±0.0	2.38±0.01	2.08±0.02
RA1700169	S19-3_CFF	35.35±0.95	21.20±0.59	18.10±0.20	7.47±0.01	5.81±0.16	2.35±0.06	3.39±0.09	2.72±0.07
RA1700170	S19-3_UoN	39.01±0.43	18.33±0.15	21.58±0.27	4.24±0.02	4.38±0.04	1.45±0.02	3.23±0.11	3.44±0.03
RA1700171	Situraja2	38.54±0.32	23.08±0.19	17.44±0.34	6.73±0.06	4.44±0.04	2.11±0.02	2.42±0.15	2.22±0.18

RA1700172	Songkhla-1	37.74±0.06	21.28±0.01	18.30±0.03	6.51±0.02	6.07±0.02	2.13±0.01	2.05±0.01	2.40±0.02
RA1700173	Songkhla-1-38	37.39±1.40	21.79±1.00	18.86±0.23	6.24±0.02	5.37±0.18	2.03±0.007	1.67±0.06	2.30±0.22
RA1700174	Songkhla-1-72	36.11±1.49	22.43±0.94	18.82±0.24	6.66±0.08	5.47±0.24	2.05±0.09	1.69±0.08	2.26±0.09
RA1700175	Songkhla-1-90	37.66±0.08	20.87±0.04	18.52±0.07	6.81±0.01	6.16±0.09	2.24±0.02	1.93±0.04	2.37±0.02
RA1700176	Sukuraja-2	37.27±0.07	22.66±0.06	17.53±0.04	7.73±0.03	4.64±0.05	2.46±0.01	2.22±0.03	2.08±0.0
RA1700177	Thung yang dang	36.26±0.02	22.85±0.01	18.86±0.04	6.25±0.01	5.30±0.01	2.00±0.01	2.70±0.00	2.35±0.00
RA1700178	Tiga Nicaru	40.68±0.62	18.57±0.30	21.49±0.35	5.59±0.06	4.03±0.01	1.83±0.02	1.50±0.08	2.91±0.02
RA1700179	TVSU738	40.47±0.03	20.08±0.07	19.17±0.10	8.27±0.05	4.03±0.03	2.21±0.02	1.05±0.00	1.90±0.020
RA1700180	TVSU89	38.88±0.17	21.74±0.52	18.62±0.09	7.77±0.03	3.81±0.03	2.30±0.02	1.59±0.01	3.01±0.02
RA1700181	TVSu880	43.20±0.01	18.26±0.06	21.44±0.05	3.29±0.02	4.23±0.05	1.20±0.02	3.25±0.05	3.19±0.12
RA1700182	TVSu879	42.83±0.03	18.89±0.02	20.64±0.10	3.49±0.00	4.83±0.02	1.25±0.00	2.51±0.02	3.50±0.01
RA1700183	TVSu702	40.52±0.06	19.32±0.03	21.59±0.03	4.51±0.01	5.44±0.01	1.60±0.00	2.84±0.00	2.22±0.00
RA1700184	TVSU 334	43.92±0.14	19.99±0.08	18.74±0.06	4.48±0.02	3.95±0.01	1.54±0.00	2.16±0.01	2.93±0.01
RA1700185	TVSU 922	42.43±0.02	20.19±0.00	19.93±0.02	3.31±0.00	4.43±0.00	1.18±0.00	2.99±0.00	3.35±0.00
RA1700186	TVSU 924	41.09±0.01	20.73±0.70	19.08±0.01	4.47±0.00	4.92±0.00	1.52±0.01	3.10±0.00	2.87±0.57
RA1700187	TVSu 323	45.09±0.00	20.69±0.04	19.30±0.02	2.85±0.00	3.71±0.00	1.05±0.00	1.67±0.00	3.57±0.00
RA1700188	TVSU1023	44.44±0.06	15.89±0.03	20.71±0.03	4.29±0.00	4.99±0.00	1.49±0.00	2.91±0.00	2.54±0.00
RA1700189	TVSU1033	44.74±0.96	14.05±0.02	22.09±0.02	3.68±0.00	4.29±0.01	1.29±0.00	2.92±0.02	3.63±0.00
RA1700190	TVSU 1231	48.286±0.04	14.57±0.01	20.87±0.01	3.59±0.00	3.70±0.01	1.27±0.00	1.39±0.00	4.64±0.00
RA1700191	TVSU702-2018	39.996±0.10	18.36±0.24	20.95±0.03	5.21±0.07	4.58±0.05	1.68±0.02	2.55±0.04	3.50±0.11
RA1700192	TVSU879-2018	40.892±0.17	18.26±0.07	20.38±0.04	5.08±0..02	4.81±0.03	1.74±0.02	1.90±0.02	4.16±0.09
RA1700193	TVSU880-2018	40.680±0.25	16.45±0.05	21.39±0.14	5.00±0.00	4.20±0.03	1.75±0.01	2.47±0.2	4.99±0.03
RA1700194	Uniswa Red	36.312±0.12	22.14±0.02	17.89±0.18	6.66±0.01	6.70±0.18	2.15±0.03	2.49±0.10	2.44±0.04
RA1700195	Uniswa Red _AB_CFF	36.184±0.28	21.70±0.11	16.86±0.05	6.85±0.01	7.05±0.13	2.26±0.00	2.85±0.02	2.65±0.15
RA1700196	Uniswa Red_Red_UoN	38.525±0.05	21.76±0.04	19.33±0.05	3.76±0.01	5.67±0.00	1.32±0.01	3.27±0.0	2.40±0.02
RA1700197	Uniswa Red_Green_UoN	41.422±0.09	17.10±0.04	22.06±0.05	3.10±0.01	3.89±0.03	1.17±0.00	3.24±0.01	4.00±0.01
RA1700198	URUG2(1)	33.897±0.38	25.32±0.16	18.17±0.02	6.82±0.01	5.47±0.06	2.05±0.03	2.70±0.03	1.80±0.02
RA1700199	Zebra Cream-8	38.444±0.10	20.84±0.03	17.57±0.05	9.41±0.01	4.87±0.05	2.68±0.02	1.89±0.04	2.76±0.01
RA1700200	Zebra Cream-10 (KM10)	39.420±0.03	18.58±0.02	18.92±0.23	8.54±0.40	5.01±0.03	2.62±0.00	1.91±0.01	3.03±0.01
Overall Min		31.95	14.04	15.87	2.43	3.17	0.90	1.05	1.48

Overall Max	48.33	29.29	23.56	11.43	7.39	3.31	4.83	5.02
Overall Mean	38.02	20.85	18.68	6.78	5.25	2.18	2.53	2.65
Overall standard deviation	3.29	3.48	1.65	1.78	0.91	0.49	0.85	0.67
Significance of line differences	**	**	**	**	**	**	**	**

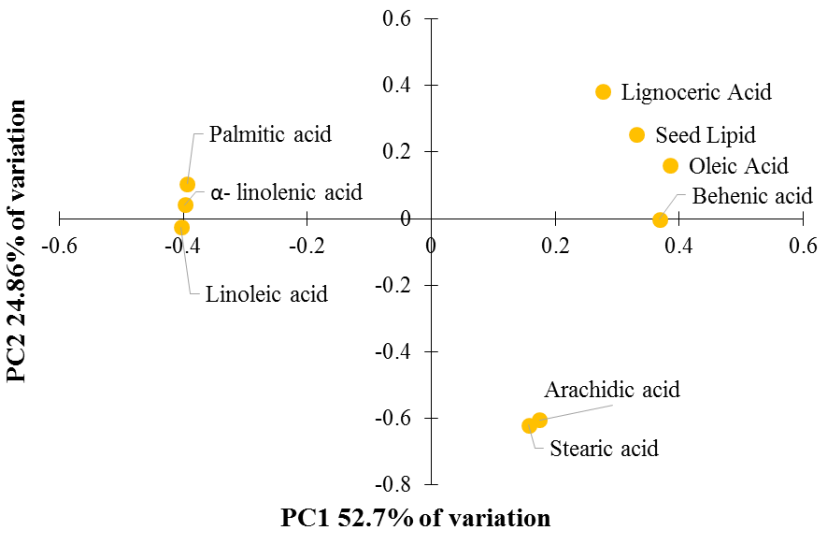

Figure S1: Principal Component Analysis (PCA) loading plot for seed lipid concentration (g/100g dry weight) and eight fatty acids for the global diversity set of 100 bambara groundnut lines. Input data were mean values calculated from three technical replicates expressed as % of total fatty acid (Table S3). PCA conducted on the correlation matrix.

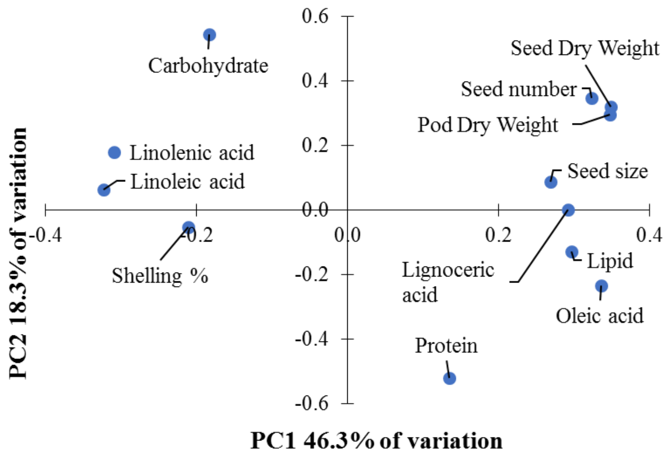

Figure S2: PCA loading plot of agronomic (seed number, pod dry weight, seed dry weight, and seed size, shelling percentage) and nutritional traits (seed lipid, protein, carbohydrate, oleic acid, lignoceric acid, linoleic acid, and linolenic acid) selected for selection indices dataset of 40 bambara groundnut lines. Principal component analysis was conducted on the correlation matrix.

Table S3: Scenario scores calculated from SND values presented in for all 15 scenarios tested for the 5 selection indices: 1) Seed lipid 2) omega 3 & 6 fatty acids 3) seed lipid & yield 4) omega 3 & 6 fatty acids & yield components, and 5) yield. Description of each index and scenario is presented in Table 5.1. SI = Selection index, DBB=Dotted brown/black eye, LBLBE=Light brown/light brown eye.

**Line ID**	**Line name**	**Selection Index & scenario**														
		**SI 1**	**SI2**	**SI 3**				**SI 4**				**SI5**				
		**1a**	**2a**	**3a**	**3b**	**3c**	**3d**	**4a**	**4b**	**4c**	**4d**	**5a**	**5b**	**5c**	**5d**	**5e**
RA1700101	100SB16ANMAM-CA-16	1.64	-1.27	0.87	-0.23	0.65	-0.12	-2.05	-3.15	-2.26	-3.03	-1.99	-1.76	-3.09	-2.53	-4.41
RA1700102	104S-1913NAM	3.29	-1.42	4.01	3.05	4.99	5.71	-0.70	-1.66	0.28	1.00	3.42	4.06	2.46	4.78	4.54
RA1700103	109BWA1BWA-1	3.66	7.12	4.41	3.80	4.19	4.94	7.87	7.26	7.66	8.39	4.56	4.94	3.95	5.69	5.83
RA1700104	20Acc118CIV-B	-0.05	-3.04	-0.71	-1.14	-0.23	-0.89	-3.70	-4.13	-3.22	-3.8	-1.76	-1.70	-2.19	-2.35	-3.44
RA1700105	56Acc89MLI-C	2.86	1.35	3.26	2.51	3.76	4.15	1.75	1.00	2.24	2.64	1.39	2.14	0.65	2.54	2.20
RA1700106	84ACC696ZMB-2	2.46	2.58	2.99	2.04	3.55	4.06	3.10	2.15	3.65	4.18	3.75	4.47	2.81	4.99	4.57
RA1700107	99SB4-2NAM-A	6.57	1.69	7.30	6.32	7.12	7.86	2.43	1.44	2.24	2.98	4.73	5.36	3.74	6.10	5.85
RA1700108	60ACC32NGA-A	-6.13	-1.83	-6.38	-5.75	-7.00	-7.25	-2.08	-1.46	-2.70	-2.95	-3.93	-3.99	-3.30	-4.24	-3.87

RA1700109	1-76Acc390 SDN-C A-11	-6.36	-4.53	-7.09	-7.28	-6.63	-7.35	-5.25	-5.45	-4.79	-5.52	-5.84	-5.71	-6.04	-6.44	-7.36
RA1700110	91UNISRSWA-B	2.12	1.02	2.33	2.39	2.52	2.73	1.22	1.28	1.41	1.62	1.96	1.93	2.02	2.14	2.41
RA1700111	92AHM968NAM-C	1.92	1.39	2.04	1.41	2.45	2.58	1.52	0.88	1.93	2.05	2.10	2.68	1.46	2.80	2.30
RA1700114	BC31107	1.20	5.13	0.58	2.39	-0.80	-1.42	4.51	6.32	3.13	2.52	-0.31	-0.54	1.49	-1.16	0.03
RA1700116	BCGC 23107	3.69	-3.76	3.15	3.87	1.91	1.38	-4.30	-3.58	-5.53	-6.06	-1.19	-1.31	-0.48	-1.84	-1.66
RA1700118	BH 16107	2.11	-0.32	1.59	3.32	0.12	-0.39	-0.84	0.88	-2.30	-2.83	-0.67	-0.90	1.06	-1.42	-0.22
RA1700120	BD	6.66	0.62	7.00	8.02	6.71	7.05	0.96	1.99	0.68	1.02	4.88	4.60	5.91	4.94	6.31
RA1700239	Burkina_2016	-5.53	0.56	-6.34	-7.36	-6.18	-6.99	-0.25	-1.28	-0.09	-0.90	-4.35	-4.39	-5.37	-5.20	-7.04
RA1700123	Cikur 2.1	3.20	-2.73	2.75	3.36	3.51	3.06	-3.18	-2.57	-2.42	-2.87	0.59	0.51	1.20	0.07	0.23
RA1700126	Cream Grey Eye-49	-2.34	-2.98	-3.00	-3.19	-3.37	-4.03	-3.64	-3.83	-4.02	-4.67	-4.41	-4.41	-4.61	-5.07	-5.93
RA1700127	Cream Light Brown Eye	-5.86	0.58	-6.64	-7.05	-7.47	-8.25	-0.19	-0.60	-1.02	-1.80	-5.19	-5.28	-5.60	-6.06	-7.25
RA1700129	DipC_CFF	-1.85	-0.95	-2.54	-2.80	-3.79	-4.48	-1.63	-1.90	-2.89	-3.58	-3.88	-3.89	-4.14	-4.58	-5.53
RA1700131	DodR_CFF	1.03	2.21	1.88	1.97	2.69	3.54	3.06	3.15	3.86	4.72	3.43	3.33	3.52	4.18	5.12
RA1700133	DBB	-6.24	2.81	-6.97	-6.70	-7.47	-8.20	2.09	2.35	1.58	0.85	-4.57	-4.70	-4.30	-5.43	-5.89
RA1700135	Exsokoto-39	-7.29	-2.53	-7.95	-6.70	-9.08	-9.74	-3.18	-1.93	-4.31	-4.97	-5.13	-5.33	-3.88	-5.99	-5.40
RA1700146	GH37107	9.72	-0.79	11.90	11.07	13.05	15.24	1.39	0.56	2.54	4.73	10.10	9.68	9.26	11.87	13.22
RA1700147	GHC36105	6.87	12.38	9.81	8.06	10.32	13.27	15.3	13.5	15.84	18.79	11.70	11.28	9.94	14.23	15.41
RA1700151	IITA686_CFF_2016	0.05	6.54	-0.54	-0.95	-1.20	-1.80	5.94	5.54	5.284	4.69	0.93	1.05	0.53	0.46	-0.54
RA1700153	IPB Bam-1	10.81	2.59	12.70	13.11	12.24	14.13	4.48	4.89	4.02	5.92	10.39	9.56	10.80	11.46	13.76
RA1700154	IPB Bam-2	10.83	3.98	13.60	12.48	13.47	16.24	6.75	5.63	6.62	9.40	11.68	11.06	10.55	13.83	15.48
RA1700155	IPB Bam-6	3.44	3.26	2.98	3.34	2.67	2.21	2.81	3.17	2.504	2.05	2.21	2.18	2.57	1.73	1.63
RA1700160	Kaaro-66	-7.31	0.86	-7.99	-7.79	-8.66	-9.34	0.18	0.38	-0.48	-1.16	-4.98	-5.05	-4.78	-5.75	-6.22
RA1700164	LunT_CFF	0.04	-4.59	0.89	0.24	2.98	3.82	-3.75	-4.39	-1.66	-0.81	1.16	1.80	0.52	2.65	2.85
RA1700165	LBLBE	-5.75	2.98	-6.49	-6.82	-7.56	-8.29	2.25	1.92	1.176	0.44	-4.31	-4.36	-4.64	-5.09	-6.15
RA1700166	Mottle Black_2016	-1.02	12.79	-1.66	-1.15	-2.45	-3.09	12.16	12.67	11.36	10.73	-0.43	-0.54	0.077	-1.18	-1.30
RA1700167	Nav4-13	-5.89	4.76	-6.55	-6.00	-7.47	-8.14	4.09	4.64	3.17	2.50	-3.53	-3.67	-2.98	-4.34	-4.45
RA1700168	Rajap 3.2	0.34	-1.58	0.81	2.29	3.15	3.62	-1.11	0.36	1.23	1.70	2.50	2.06	3.98	2.53	4.47
RA1700169	S19-3	-3.37	-1.29	-4.21	-3.17	-1.57	-2.41	-2.12	-1.08	0.52	-0.31	-0.90	-1.12	0.14	-1.96	-1.76
RA1700172	Songhkla1	-4.35	-3.07	-4.99	-5.62	-3.66	-4.30	-3.71	-4.34	-2.38	-3.02	-3.44	-3.27	-4.06	-3.91	-5.18
RA1700180	TVSU89	-0.79	4.47	-1.48	-0.41	-2.26	-2.95	3.78	4.85	2.99	2.31	-2.00	-2.19	-0.93	-2.88	-2.50
RA1700194	Uniswa Red UR343	-1.88	-1.69	-2.49	-1.71	-1.82	-2.43	-2.30	-1.52	-1.64	-2.24	-1.89	-2.03	-1.12	-2.63	-2.46
RA1700200	Zebra Cream-10	-3.64	4.19	-4.03	-4.40	-4.60	-4.99	3.81	3.44	3.233	2.85	-2.60	-2.34	-2.97	-2.72	-3.48

Table S4: Scenario scores for the bambara groundnut lines with the highest 15 scores for scenario with each selection index. Description of each scenario tested within each index is as described in Table 5.1. One scenario was tested each for selection index 1 and 2; four scenarios were tested for selection index 3 and five scenarios were tested for selection index 5. For selection index 3 and 4, the two scenarios with the highest scores are shown. For selection index 5, the three scenarios with the highest scores are shown. DBB=Dotted brown/black eye, LBLBE=Light brown/light brown eye.

**Selection index 1: Seed lipid**		**Selection index 2: Omega 3 & 6 fatty acid**		**Selection index 3: Seed lipid & yield**		
Scenario: Oleic acid<br>***Traits selected:*** Oleic acid		***Scenario:*** Linoleic and linolenic acid<br>***Traits selected:*** Linoleic and linolenic acid		***Scenarios:*** a) Oleic acid & 1 yield component<br>b) Oleic acid & 2 yield components<br>***Traits selected:*** a) Oleic acid, seed weight<br>b) Oleic acid, seed number, seed weight		
Bambara groundnut line	*Scenario score*	*Bambara groundnut line*	*Scenario score*	*Bambara groundnut line*	*Scenario score (3a)*	*Scenario score (3b)*
IPB Bam-2	10.83	Mottle Black_2016	12.79	IPB Bam-2	13.60	16.25
IPB Bam-1	10.81	GHC36105	12.38	IPB Bam-1	12.70	14.14
GHC36105	6.87	BC31107	5.13	GHC36105	9.81	13.27
BD	6.66	Nav4-13	4.76	99SB4-2NAM-A	7.30	7.86
99SB4-2NAM-A	6.57	TVSU89	4.47	BD	7.00	7.06
BCGC 23107	3.69	Zebra Cream-10	4.19	104S-1913NAM	4.01	5.71
109BWA1BWA-1	3.66	IPB Bam-2	3.98	109BWA1BWA-1	4.41	4.94
IPB Bam-6	3.44	IPB Bam-6	3.26	LunT_CFF	0.89	3.82
104S-1913NAM	3.29	LBLBE	2.98	DodR_CFF	1.88	3.55
Cikur 2.1	3.20	DBB	2.81	Cikur 2.1	2.75	3.06
56Acc89MLI-C	2.86	IPB Bam-1	2.59	91UNISRSWA-B	2.33	2.72
91UNISRSWA-B	2.12	DodR_CFF	2.21	92AHM968NAM-C	2.04	2.58
BH 16107	2.11	99SB4-2NAM-A	1.69	IPB Bam-6	2.98	2.22
92AHM968NAM-C	1.92	92AHM968NAM-C	1.39	BCGC 23107	3.15	1.38
100SB16ANMAMCA16	1.64	56Acc89MLI-C	1.35	100SB16ANMAMCA16	0.87	-0.12

Selection index 4: Omega 3 & 6 Fatty acid, yield

Scenarios: a) Linoleic, linolenic acid & 1 yield component
b) Linoleic, linolenic acid & 2 yield component

Selection index 5: Yield

Scenarios: a) 1 yield component
b) 2 yield component
c) 3 yield component

Traits selected:	a) Linoleic, linolenic acid, seed weight<br>b) Linoleic, linolenic acid, seed weight, seed number		***Traits selected:***	a) Seed weight<br>b) Seed weight & number<br>c) Seed weight, seed number & seed size		
Bambara groundnut line	*Scenario score (4a)*	*Scenario score (4b)*	*Bambara groundnut line*	*Scenario score (5a)*	*Scenario score (5b)*	*Scenario score (5c)*
GHC36105	15.33	18.79	GHC36105	11.70	14.23	15.41
Mottle Black_CFF_2016	12.16	10.73	IPB Bam-2	11.68	13.83	15.48
109BWA1BWA-1	7.87	8.40	IPB Bam-1	10.39	11.46	13.76
IPB Bam-2	6.75	9.40	BD	4.88	4.94	6.31
IITA686_CFF_2016	5.94	4.69	99SB4-2NAM-A	4.73	6.10	5.85
BC31107	4.51	2.52	109BWA1BWA-1	4.56	5.69	5.83
IPB Bam-1	4.48	5.92	DodR_CFF	3.43	4.18	5.12
Nav4-13	4.09	2.50	104S-1913NAM	3.42	4.78	4.54
Zebra Cream-10	3.81	2.85	IPB Bam-6	2.21	1.73	1.63
TVSU89	3.78	2.31	92AHM968NAM-C	2.10	2.80	2.30
DodR_CFF	3.06	4.72	91UNISRSWA-B	1.96	2.14	2.41
IPB Bam-6	2.81	2.05	LunT_CFF	1.16	2.65	2.85
99SB4-2NAM-A	2.43	2.98	56Acc89MLI-C	1.39	2.54	2.20
LBLBE	2.25	0.44	IITA686_CFF_2016	0.93	0.46	-0.54
Dotted Brown black eye	2.09	0.85	Cikur 2.1	0.59	0.07	0.23
